# The Effect of Fluid Intake Following Dehydration on Subsequent Athletic and Cognitive Performance: a Systematic Review and Meta-analysis

**DOI:** 10.1186/s40798-017-0079-y

**Published:** 2017-03-18

**Authors:** Danielle McCartney, Ben Desbrow, Christopher Irwin

**Affiliations:** School of Allied Health Sciences and Menzies Health Institute Queensland, Griffith University, Gold Coast, Australia

**Keywords:** Athletic, Cognitive, Performance, Mood, Dehydration, Fluid intake

## Abstract

**Background:**

The deleterious effects of dehydration on athletic and cognitive performance have been well documented. As such, dehydrated individuals are advised to consume fluid in volumes equivalent to 1.25 to 1.5 L kg^−1^ body mass (BM) lost to restore body water content. However, individuals undertaking subsequent activity may have limited time to consume fluid. Within this context, the impact of fluid intake practices is unclear. This systematic review investigated the effect of fluid consumption following a period of dehydration on subsequent athletic and cognitive performance.

**Methods:**

PubMed (MEDLINE), Web of Science (via Thomas Reuters) and Scopus databases were searched for articles reporting on athletic (categorized as: *continuous*, *intermittent, resistance*, *sport-specific* and *balance* exercise) or cognitive performance following dehydration of participants under control (no fluid) and intervention (fluid intake) conditions. Meta-analytic procedures determined intervention efficacy for continuous exercise performance.

**Results:**

Sixty-four trials (*n* = 643 participants) derived from 42 publications were reviewed. Dehydration decreased BM by 1.3–4.2%, and fluid intake was equivalent to 0.4–1.55 L kg^−1^ BM lost. Fluid intake significantly improved continuous exercise performance (22 trials), Hedges’ *g* = 0.46, 95% CI 0.32, 0.61. Improvement was greatest when exercise was performed in hotter environments and over longer durations. The volume or timing of fluid consumption did not influence the magnitude of this effect. Evidence indicating a benefit of fluid intake on *intermittent* (10 trials), *resistance* (9 trials), *sport-specific* (6 trials) and *balance* (2 trials) exercise and on cognitive performance (15 trials) was less apparent and requires further elucidation.

**Conclusions:**

Fluid consumption following dehydration may improve continuous exercise performance under heat stress conditions, even when the body water deficit is modest and fluid intake is inadequate for complete rehydration.

**Electronic supplementary material:**

The online version of this article (doi:10.1186/s40798-017-0079-y) contains supplementary material, which is available to authorized users.

## Key Points

1. Evidence indicating a beneficial effect for fluid intake on athletic performance was strongest when a continuous exercise task was employed; whereas limited research has examined the effect of fluid intake on intermittent, resistance and sport-specific exercise performance.
2. The magnitude of improvement was greater when the continuous exercise was performed at elevated environmental temperatures and over longer exercise durations.
3. Whilst the volume of fluid consumed (relative to BM lost) did not appear to influence the size of the treatment effect, fluid intake at levels that comply with current recommendations for restoring body water content (1.25–1.50 L kg BM lost^−1^) are yet to be thoroughly investigated.

## Background

The deleterious effects of dehydration (fluid loss) on athletic and cognitive performance have been extensively researched. Recent meta-analyses detected meaningful decrements in aerobic [1] and anaerobic [2] exercise performance and muscular strength and endurance [2] when subjects commenced activity in an already dehydrated state. Experimental investigations have also demonstrated motor-skill impairments on sport-specific exercise tests (e.g. cricket [3], basketball [4, 5], golf [6], field hockey [7] and surfing [8]) following fluid loss. Whilst evidence indicating a detrimental effect of dehydration on cognitive function is less consistent [9], decline in memory, perceptual discrimination and mood state has been observed in some studies [10]. Dehydration is commonly observed amongst athletes [11–14] and manual workers (e.g. military, fire fighters and labourers) [15], who rely upon physical and mental proficiencies to compete or train at elite levels and remain productive in the workforce. This evidence has provided the rationale for fluid replacement recommendations.

The American College of Sports Medicine (ACSM) Guidelines on Exercise and Fluid Replacement [16] and the Position of the Academy of Nutrition and Dietetics on Nutrition and Athletic Performance [17] recommend dehydrated individuals consume 1.25 to 1.50 L of fluid per kilogram of body mass (BM) lost to replenish body water content, if the fluid deficit is large and recovery time is limited (i.e. <12 h). Whilst the importance of returning to euhydration over a period of a day(s) is not in dispute, many individuals are required to undertake repeated bouts of activity, where limited time between tasks exists or the demands of a subsequent activity (i.e. type, duration and intensity) and/or the environment (e.g. conflict zone) may influence the appropriateness of the aforementioned guidelines. Within this context, consuming fluid has the potential to enhance or inhibit performance. Thus, determining rehydration strategies that counteract the detrimental effects of fluid loss, whilst optimizing performance on subsequent tasks, is important.

Ingesting large volumes of fluid may cause gastrointestinal (GI) discomfort, impeding performance. Particularly if the amount of time available to consume fluid is limited or fluids with higher calorie loads (e.g. milk-based beverages) and hence slower rates of gastric emptying are ingested [18, 19]. The nature of the subsequent activity, e.g. the mechanical ‘bouncing’ action caused by high intensity running, may also impact GI symptomology [20]. Conversely, drinking large fluid volumes promotes rapid initial gastric emptying [19], facilitating fluid absorption, and may convey greater benefit than drinking smaller volumes. To date, the majority of investigations examining the effect of ingested fluid volume on subsequent performance have employed a prolonged (i.e. overnight) rehydration period [21–25], reducing the probability of GI disturbance influencing subsequent performance. Thus, the importance of ingested fluid volume and its impact on subsequent exercise performance outcomes remains unclear. The aim of the present systematic review and meta-analysis was to examine the impact of consuming fluid following a period of dehydrating sweat loss on subsequent athletic and cognitive performance. Understanding how to maximize the benefits of fluid intake under these circumstances will inform the development of future fluid replacement guidelines.

## Methods

The following research protocol was devised in accordance with specifications outlined in the *Preferred Reporting Items for Systematic Reviews and Meta-Analysis Protocols PRISMA-P 2015 Statement* [26]. The methodology of this review is registered at the International Prospective Register for Systematic Reviews, identification code CRD42016036560.

### Literature Search

**Fig. 1:**
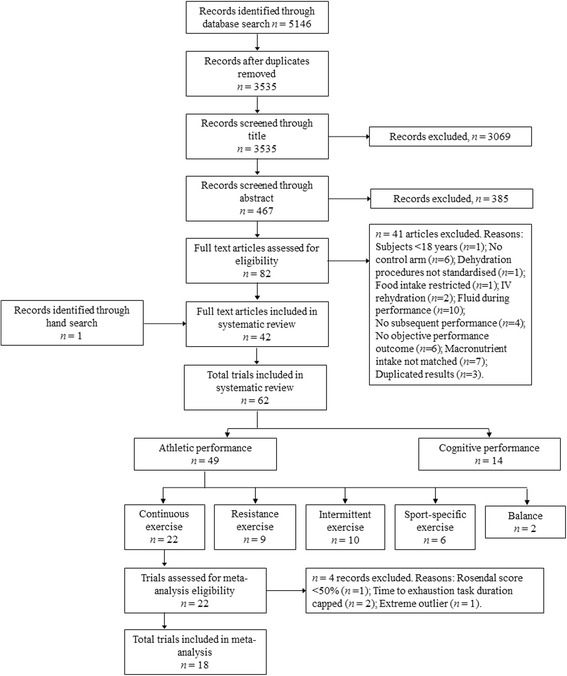
PRISMA Flow Chart (study selection methodology). Where a study contained >1 intervention-arm that was eligible for inclusion (i.e. paired against a suitable control condition), these were treated as separate ‘studies’ termed ‘*trials*’

Potential research studies were identified by searching the online databases PubMed (MEDLINE), Web of Science (via Thomas Reuters) and Scopus from inception until April 2016 using the terms exercise, athletic, performance, mood and cognit* (the symbol was used to capture all words beginning with cognit, e.g. cognitive, cognition), each in combination with “fluid replacement” (the enclosed quotation marks were used to search for an exact phrase), “fluid ingestion”, “fluid intake”, “fluid consumption”, “fluid administration”, rehydrat* and euhydrat*. Records that contained irrelevant terms (patient, rat, mouse, aged care, reaction, disease, illness, bacteria, children and elderly) were excluded from the literature search using the Boolean search operator ‘NOT’. Two investigators (D.M. and C.I.) independently screened potential research studies to identify relevant texts. Full details of the screening process are presented in Fig. 1. Initially, all irrelevant titles were discarded. The remaining studies were systematically screened for eligibility by abstract and full text, respectively. The final decision to include or discard research studies was made between two investigators (D.M. and C.I.), with any disagreement resolved in consultation with a third investigator (B.D.). The reference lists of all included studies were then hand searched for missing publications.

### Inclusion and Exclusion Criteria

Research studies containing a control-arm and one or more intervention-arms fulfilling the following criteria were eligible for inclusion:

1. Repeated measures experimental design.
2. Human studies on adult (≥18 years of age) male or female participants with no known medical conditions or co morbidities.
3. An athletic or cognitive performance outcome (see “Primary and Secondary Research Outcomes” section below for full description) was measured under control and intervention conditions. The control condition was dehydration with no fluid or negligible fluid intake, where ‘negligible’ fluid intake was accepted as ≤200 mL. This threshold was intended to broaden the inclusion criteria, allowing greater data capture and increased statistical power, since this was the first review to examine the effect of fluid intake on subsequent athletic and cognitive performance. The intervention condition was defined as dehydration with concurrent and/or subsequent fluid intake >200 mL.
4. The mode of dehydration was standardized, i.e. all participants were subjected to the same dehydration protocol, with or without fluid intake, on intervention and control trials.
5. Hydration status was manipulated before the performance task commenced, i.e. dehydration and fluid ingestion occurred before, not during, the performance assessment. A schematic representation of the experimental protocol is displayed in Fig. 2.

**Fig. 2:**
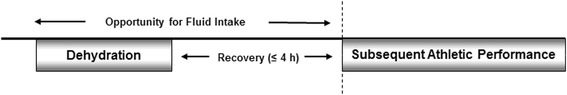
A schematic representation of the experimental protocol employed in studies eligible for inclusion in the present review
6. There is ‘limited’ time to consume fluid, defined as: ≤4 h between completing the dehydration protocol and commencing the subsequent performance test, unless performance followed an overnight fasting period.
7. An objective measurement of hydration status (e.g. body weight, urine specific gravity, plasma or urine osmolality or plasma volume) was used to indicate the level of dehydration attained.
8. Accessible full text articles written in English.

Studies were excluded from the review if: (1) dehydration involved restriction of food intake; (2) fluids were not administered orally (e.g. intravenous infusions) or (3) were co-administered with another experimental treatment (e.g. glycerol, l-alanyl-l-glutamine or external cooling); (4) subjects ingested >200 mL of fluid or an unspecified volume of fluid on control trials (e.g. Bardis et al. [27] and Baker et al. [28]); (5) macronutrient intake was not matched on experimental trials or (6) performance data was not adequately reported, i.e. values were not quantified, or descriptive terms were not used.

For the purpose of this systematic review, research studies containing multiple intervention-arms that were eligible for inclusion (each paired against a suitable ‘no fluid’ control group) (e.g. McConell et al. [25] tested athletic performance under two different fluid conditions and Hillman et al. [29] tested athletic performance under different environmental conditions) were treated as separate experimental studies termed ‘*trials*’. Separate trials derived from a single research study are denoted by additional letters (i.e. a–d) in the citation.

### Methodological Quality Assessment

All eligible studies were examined for publication bias using the Rosendal Scale [30]. Excellent methodological quality is indicated by a Rosendal Score ≥60% [31]. Items 7, 8 and 9 of the scale, pertaining to the use of blinding procedures, were omitted from the evaluation as oral fluid ingestion cannot be blinded. Scoring was determined by dividing the number of ‘yes’ responses by the total number of applicable items and reported for all included studies. Studies were excluded from meta-analyses if they received a Rosendal score <50%.

### Data Extraction and Synthesis

Data were extracted from relevant publications following the Cochrane Handbook for Systematic Reviews of Interventions *Checklist of Items to Consider in Data Collection or Data Extraction* [32] and entered into a Microsoft Excel spread sheet.

#### Primary and Secondary Research Outcomes

The first primary research outcome was (1) objective indicators of athletic performance; subjective measurements of performance (e.g. ratings of perceived exertion) were not examined in this review. The types of athletic performances studied were broadly classified as follows: (a) continuous exercise; (b) intermittent exercise; (c) resistance exercise; (d) sport-specific exercise and (e) balance tasks. Performances that incorporated a coordinated motor-movement resembling some skill involved in a particular sporting event were categorized as ‘sport-specific’ exercises, whereas non-specific sporting activities (e.g. sprint running) were categorized into one of the remaining groups (where possible). Where more than one type of athletic performance was measured within a single experimental trial (e.g. Walsh et al. [33] examined performance on continuous and resistance exercise tasks), the performances were presented in their respective categories and treated as separate trials. The second primary research outcome was (2) objective indicators of cognitive function, including subjective measurements of mood state. The decision to include mood as a primary research outcome was based on previous suggestions that mood and symptom questionnaires may be more sensitive to subtle changes in hydration status than tests of cognitive ability [34]. Subjective ratings of GI discomfort and thirst following fluid ingestion were intended as the secondary research outcomes. However, very few investigations evaluated GI symptomology [25] or thirst [35–37]. Thus, insufficient data were available to complete secondary analyses.

#### Other Relevant Data

Other information extracted from relevant research studies included

1. Participant characteristics: description, age, euhydrated body mass (BM) and maximal oxygen consumption (VO_2 max_)
2. The dehydration protocol: mode of dehydration, ambient temperature and relative humidity, protocol duration, level of dehydration and time from finishing the dehydration protocol to receiving fluid
3. The rehydration protocol: fluid type, volume of fluid consumed, drink time and time from finishing fluid to commencing performance task
4. Performance task: task description and performance outcomes, ambient temperature, relative humidity, rate of airflow, intensity and duration, where applicable

Percentage of BM loss was used to indicate the level of dehydration attained. If the percentage of BM loss was not directly reported, values were calculated from euhydrated BM (kg) and BM mass loss (kg) using the following formula:

$$ \%\mathrm{B}\mathrm{M}\;\mathrm{lost}=\frac{\mathrm{BM}\kern0.24em \mathrm{loss}\kern0.24em \left(\mathrm{kg}\right)}{\mathrm{Euhydrated}\kern0.24em \mathrm{BM}\kern0.24em \left(\mathrm{kg}\right)}\times 100 $$

The volume of fluid consumed was expressed as a proportion of BM loss, i.e. relative fluid intake (L kg BM lost^−1^). If the quantity of fluid consumed was not expressed as a proportion of BM loss, values were calculated from fluid intake (L) and percentage of BM loss using the following formula:

$$ \mathrm{L}\cdot \mathrm{kg}\;\mathrm{B}\mathrm{M}\;{\mathrm{lost}}^{\hbox{-} 1}=\frac{\mathrm{Fluid}\kern0.24em \mathrm{intake}\kern0.24em \left(\mathrm{L}\right)}{\left(\%\mathrm{BM}\;\mathrm{loss}\times 0.01\right)\times \mathrm{euhydrated}\;\mathrm{BM}\;\left(\mathrm{kg}\right)}\times 100 $$

If the volume of fluid consumed was unknown, the BM deficit post-rehydration has been reported.

Time from completing the dehydration protocol to commencing the subsequent performance task (*recovery time*) and time from commencing fluid ingestion to commencing the subsequent performance task (*fluid assimilation time*) were approximated from the experimental protocol, where adequate information was provided.

If necessary information was not available from the published article and it was published within the previous 10 years, authors were contacted via email with a request to provide missing data.

### Statistical Analyses

Sufficient data were available to perform a meta-analysis examining the impact of fluid consumption following a period of dehydration on subsequent continuous exercise performance. Meta-analyses were not performed on other types of athletic performance or cognitive function because: (1) intermittent and sport-specific exercise performance trials were methodologically heterogeneous, particularly in regards to the exercise protocol and outcomes used to determine a treatment effect; (2) few authors responded to an email request for raw data regarding resistance exercise performance, preventing computation of the correlation coefficient and (3) cognitive performance data was rarely quantified (descriptive terms only).

#### Meta-analysis on Continuous Exercise Performance

All statistical procedures were performed using IBM SPSS Statistical Software, Version 22.0 and Comprehensive Meta-Analysis, Version 3.0. Repeated measures intervention effect sizes were calculated as Hedges’ *g* [38], where the mean difference between each intervention and control performance score was standardized against the SD of the performance change and corrected for bias due to small sample size. The magnitude of effect was defined in accordance with Cohen [39]: ES ≤0.2 = small; ≥0.5 ES >0.2 = medium and ≥0.8 = large, where a positive Hedges’ *g* value indicates a beneficial effect of fluid intake on continuous exercise performance. Where the SD of the performance change was not reported, the missing value was imputed using a correlation coefficient [32] calculated with the following formula:

$$ \mathrm{S}{\mathrm{D}}_{\Delta}=\sqrt{\left({\mathrm{SD}}_{\mathrm{No} \mathrm{F}\mathrm{l}\mathrm{u}\mathrm{i}\mathrm{d}}^2+{\mathrm{SD}}_{\mathrm{Fluid}}^2\right) -\left(2\times \mathrm{R}\times {\mathrm{SD}}_{\mathrm{No} \mathrm{F}\mathrm{l}\mathrm{u}\mathrm{i}\mathrm{d}}\times {\mathrm{SD}}_{\mathrm{Fluid}}\right)} $$

Where SD_∆_ is the missing standard deviation of change and *R* is the correlation coefficient. *R* was approximated as the mean correlation coefficient (*R* = 0.84) calculated using raw performance data from nine continuous exercise trials (derived from four separate publications). Sensitivity analysis was performed using *R* = 0.50, 0.74 and 0.94 to test the robustness of the imputed correlation coefficient. The weighted mean treatment effect was calculated using random-effect models, where trials were weighted by the inverse variance for the standardized performance change. Statistical significance was attained if the 95% CI did not include zero. Data are described as mean ± SD, unless otherwise indicated; articles that reported SEM had their values multiplied by the square root of the sample size to convert to SD. All research studies measuring performance on a continuous exercise task used a single objective measurement to demonstrate the presence or absence of a treatment effect (e.g. time to exhaustion or power output). Hence, no additional precautions were taken to limit data dependency.

#### Heterogeneity and Sensitivity Analyses

Heterogeneity was assessed using Cochran’s Q and the *I* ^2^ index. Low, moderate and high heterogeneity was indicated by an *I* ^2^ value of 25, 50 and 75%, respectively [40]. A *p* value <0.10 for Cochran’s Q was used to indicate significant heterogeneity [32]. Sensitivity analyses were performed by removing individual trials and examining the effect of each study on the results of the weighted mean treatment effect.

#### Meta-regression Analysis

A priori, we identified the volume of fluid ingested (L kg^−1^ BM lost) and fluid assimilation time as variables that might moderate the effect of fluid intake on athletic performance. However, prior research indicates that environmental temperature, exercise duration and the ecological validity of the exercise protocol employed may influence the effect of dehydration on athletic performance [29, 41–44], as might level of fluid loss (% BM loss) incurred. Therefore, we explored the relationship between these variables and the magnitude of the treatment effect (Hedges’ *g*) using a restricted maximum likelihood multiple meta-regression (random effects) model that controlled for potential confounders. Restricted maximum likelihood simple meta-regression was also performed to explore the influence of environmental temperature on Hedges’ *g* values. The ecological validity of each continuous exercise protocol was defined in accordance with Goulet [43], where fixed-power time to exhaustion (TTE) exercise protocols were considered non-ecologically valid and time-trial type exercise protocols (including protocols measuring work completed within a set timeframe) were classified as ecologically valid. Exercise duration was taken as the mean total exercise time (min) for control and intervention trials. One study did not report total exercise time [29], therefore exercise duration was approximated as per Stewart et al. [37], who performed a comparable performance test.

As per Savoie et al. [2], regression analyses were examined for influential cases and outliers (studentized residuals and cook’s distance). Tests for normality of residuals (Shapiro-Wilk test), multicollinearity (variance inflation factor, VIF), autocorrelation (Durbin-Watson statistic), homoscedasticity and linearity of the relationship between dependent and independent variables (plot of residuals versus predicted values) ensured that analyses did not violate assumptions of meta-regression. Statistical significance was accepted as *p* < 0.05.

#### Systematic Review

All athletic and cognitive performances are presented in the systematic review investigating the effect of dehydration and fluid intake on subsequent athletic and cognitive performance. Whilst it was our intention to calculate within-subject intervention effect sizes for all athletic performance outcomes, the vast majority of the publications included in this review did not provide the necessary data to complete a paired analysis. Further, the types of performances investigated varied widely amongst studies, such that the missing SD of change could not be estimated from a known correlation coefficient. To enable comparison of effects across studies, ES were approximated as Hedges’ *g* for *independent* groups. The mean difference between each intervention and control performance score was standardized against a pooled SD and corrected for bias due to small sample size using the supplementary spreadsheet by Lakens [45]. This approach will likely underestimate the magnitude of the true effect. Cognitive performance outcomes are presented in descriptive terms only, since few publications quantified the effect of fluid intake numerically. Statistical significance was accepted as *p* < 0.05 in all studies.

## Results

### Overview of Studies and Study Quality

Sixty-four repeated measures trials (*n* = 643 healthy participants, 93% male, excluding Del Coso et al. [46] where gender was not specified, NS) derived from 42 original publications were included in the present systematic review. Methodological quality assessment yielded a median Rosendal Score of 58%. Two trials received a Rosendal Score <50% [47, 48]. Whilst these studies are presented in the systematic review, they were not deemed eligible for inclusion in subsequent meta-analysis. The highest Rosendal Score of 83% was calculated for Rodrigues et al. [49]. Complete results of the quality assessment are displayed in Additional file 1: Table S1.

### Study Characteristics

Characteristics of included studies are summarized in Tables 1, 2, 3, 4, 5 and 6. (Full details are presented in Additional file 1: Table S2, S3, S4, S5, S6 and S7). Dehydration and rehydration protocols were heterogeneous amongst included trials. In 57 out of the 61 trials reviewed, dehydration was accomplished via passive heat exposure (*n* = 11) [42, 47, 50–56] or physical activity (*n* = 47), conducted in a thermoneutral laboratory ≤25 °C (*n* = 16) [24, 25, 29, 35, 48, 55, 57–61], heated environmental chamber (*n* = 28) [22, 29, 33, 36, 37, 41, 46, 49, 62–71] or environmental conditions not specified (*n* = 3) [72, 73]. The remaining trials reduced body water content through warm water immersion (*n* = 2) [74, 75] or dietary fluid restriction in combination with 2-h moderate intensity exercise 24 h prior to testing performance (*n* = 2) [76, 77]. In 46 trials (74%) [22, 24, 29, 36, 37, 41, 42, 46, 47, 49–53, 55–59, 61–63, 66, 67, 71, 73–77], dehydration yielded BM losses ≥2%. Of these, 27 trials (43%) dehydrated participants by ≥3.0% of initial BM [22, 24, 29, 37, 41, 42, 46, 50, 52, 53, 56, 61, 62, 66, 67, 74–77]. Mean BM losses ranged from 1.3 [72] to 4.2% [41]. Ingested fluids were predominantly water or saline solution (0.05–0.50% NaCl). Studies administering carbohydrate-containing fluids were often excluded due to unequal provision of macronutrients on control and intervention trials. Two included studies failed to specify the type of fluid consumed (this was *presumed* to be water) [42, 56], and one study did not report dietary standardization procedures or specify whether subjects consumed food during the prolonged (>12 h, overnight) period of intervention [22]. The volume of fluid administered ranged from 0.40 [72] to 1.55 L kg^−1^ BM lost [65]. In 20 trials (33%), participants ingested a volume of fluid to replace <100% of sweat losses [24, 25, 33, 35, 47, 57–59, 62, 68, 70–72, 76, 77]. Only 2 trials [22, 65] provided a volume of fluid that complied with current recommendations for restoring fluid loss (1.25–1.50 L kg BM lost^−1^) [16, 17]. In 14 trials, dehydrated control subjects ingested a small volume of non-nutritive fluid (≤200 mL) [42, 55, 56, 62, 63, 66, 71] or mouth rinse [33].

**Table 1:** Characteristics of research studies evaluating athletic performance on continuous exercise tasks (studies are presented by order of effect)

Citation	Participants	VO_2 max_	Dh protocol (exercise intensity); ambient temperature; duration	Total REC (min)	Dh trial BM loss (%)	Fluid type	Fluid assimilation time (min)	Fluid intake (L kg BM lost^−1^)	Duration (min)	Performance indicator	Ambient temperature; RH; airflow	Hedges’ *g*
Kenefick et al. (2010a) [41]	8 M	43.6 ± 4.1	EX; 50 °C; 3-h W/R	120	4.1	NaCl	300	1.05	15	TW _(cycle ergometer)_	10 °C	0.05
Chevront et al. (2005a) [42]	8 (6 M), physically active	48 ± 9	HT	210	3.0	NS	390	1.00	30	TW _(cycle ergometer)_	2 °C; 50%; 2.2 m/s	0.11
Stewart et al. (2014) [37]	7 M, recreational cyclists	52.7 ± 7.9	EX (H); 37 °C; 120 min	120	3.8	Water	120	1.15	7.2	TT _(cycle ergometer)_	18–25 °C; 20–30%	0.12
McConell et al. (1999a) [25]	8 M, well-trained cyclists/triathletes	63.8 ± 1.2	EX (V); 21 °C; 45 min	0	1.9	Water	45	1.00	15	TW _(cycle ergometer)_	21 °C; 41%; airflow NS	0.15
McConell et al. (1999b) [25]	8 M, well-trained cyclists/triathletes	63.8 ± 1.2	EX (V); 21 °C; 45 min	0	1.9	Water	45	0.50	15	TW _(cycle ergometer)_	21 °C; 41%; airflow NS	0.24
McConell et al. (1997a) [24]	7 M, well-trained cyclists/triathletes	68. ± 2.5	EX (V); 21 °C, 120 min	0	3.2	Deionized water	120	0.50	3.5	TTE _(cycle ergometer)_	21 °C, 43%; airflow NS	0.24
Kenefick et al. (2006) [36]	8 M, unacclimated	63.7 ± 10.2	EX (M); 36 °C; 75 min	30	2.3	NaCl + NNS	20	1.00	55	TTE _(treadmill running)_	37 °C; 42%	0.28*
Hillman et al. (2011a) [29]	7 M, unacclimated, competitive cyclists	NS	EX (V); 34 °C; 90 min	15	3.0	Water	105	1.00	7.2	PO _(cycle ergometer)_	23 °C	0.32
Chevront et al. (2005b) [42]	8 (6 M) physically active	48 ± 9	HT	210	2.9	NS	390	1.00	30	TW _(cycle ergometer)_	20 °C; 50%; 1 m/s	0.35*
Paik et al. (2009) [50]	10 M, moderately active	53.6 ± 11.4	HT	120	3.0	Water	120	1.00	30	TTE _(treadmill running)_	NS	0.43
McConell et al. (1997b) [24]	7 M, well-trained cyclists/triathletes	68.4 ± 2.5	EX (V); 21 °C, 120 min	0	3.2	Deionized water	120	1.00	4.2	TTE _(cycle ergometer)_	21 °C, 43%; airflow NS	0.58*
Hillman et al. (2011b) [29]	7 M, unacclimated, competitive cyclists	NS	EX (V); 34 °C; 90 min	15	3.8	Water	105	1.00	7.2	PO _(cycle ergometer)_	34 °C	0.60*
Kenefick et al. (2010c) [41]	8 M	46.3 ± 5.2	EX; 50 °C; 3-h W/R	120	4.0	NaCl	300	1.05	15	TW _(cycle ergometer)_	30 °C	0.64*
Castellani et al. (1997) [62]	8 M, unacclimated	57.9 ± 4.5	EX (M); 33 °C; 180 min	135	4.1	NaCl + NNS	120	0.50	72	TTE _(treadmill walking)_	36 °C; 47%; 2.3 m/s	0.68*
Walsh et al. (1994a) [33]	6 M, endurance cyclists/ triathletes	61.4 ± 4.4	EX (V); 30 °C; 60 min	0	1.8	NaCl + NNS	50	0.90	8.2	TTE _(cycle ergometer)_	30 °C; 60%; 0.8 m/s	0.74*
Kenefick et al. (2010b) [41]	8 M	45.3 ± 4.6	EX; 50 °C; 3-h W/R	120	4.2	NaCl	300	1.05	15	TW _(cycle ergometer)_	20 °C	0.79
Kavouras et al. (2006) [77]	8 M, acclimated, endurance cyclists	61.4 ± 2.3	FR + EX (M); 120 min	>12 h	3.9	Water + NNS	80	0.75	23	TTE _(cycle ergometer)_	37 °C; 48%; 2.54 m/s	0.81*
Kenefick et al. (2010d) [41]	8 M	43.7 ± 7.0	EX; 50 °C; 3-h W/R	120	4.1	NaCl	300	1.05	15	TW _(cycle ergometer)_	40 °C	0.87*
Below et al. (1994) [63]	8 M, acclimated, endurance trained	62.9 ± 2.8	EX (V); 31 °C; 50 min	0	2.0	NaCl + NNS	50	1.00	11	TT _(cycle ergometer)_	31 °C; 54%	0.89*
Casa et al. (2000) [76]	8 M, unacclimated, endurance cyclists	61.4 ± 2.3	FR + EX (M); 120 min	>12 h	3.9	NaCl + NNS	35	0.50	27	TTE _(cycle ergometer)_	37 °C; 2.3 m/s	1.25*
Melin et al. (1994) [47]	6 M, unacclimated, endurance trained	57.5 ± 4.2	HT	60	2.6	Water	NS	0.50	97	TTE _(treadmill marching)_	35 °C; 20–30%; 0.8 m/s	1.23*
Hasegawa et al. (2006) [64]	9 M, untrained	48.5 ± 4.5	EX (M); 32 °C; 60 min	4	1.6	Water	65	1.00	4.4	TTE _(cycle ergometer)_	32 °C; 80%	4.01*

Exercise intensity is described as high (H), vigorous (V) or moderate (M), in accordance with classifications outlined by Norton et al. [86]. Values are Hedges’ *g* effect sizes

*Dh* dehydration, *EX* exercise, *FR* fluid restriction, *HT* heat, *NNS* non-nutritive sweetener, *NS* not specified, *PO* power output, *RH* relative humidity, *Total REC* time from completing the dehydration protocol to commencing the subsequent task, *TT* time trial, *TTE* time to exhaustion, *TW* total work, *W*/*R* work rest cycle

*Significant difference between performances undertaken with and without fluid replacement (*p* < 0.05)

**Table 2:** Characteristics of research studies evaluating athletic performance on intermittent exercise tasks

Citation	Participants	VO_2 max_	Dh protocol (EX intensity); ambient temperature; duration	Total REC (min)	Dh trial BM loss (%)	Fluid type	Fluid assimilation time (min)	Fluid intake (L kg BM lost^−1^)	Intermittent exercise task	Ambient temperature; RH; airflow	Performance outcomes(s)	Performance outcomes significantly affected (Hedges’ *g*)
Walsh et al. (1994b) [33]	6 M, endurance cyclists/triathletes	61.4 ± 4.4	EX (V); 30 °C; 60 min	15	1.8	NaCl + NNS	65	0.90	^a^IST	NS	Max. velocity; lower limb force	No effect
Maxwell et al. (1999) [65]	11 M, untrained	NS	EX (M); 32 °C; 48 min	120	1.5	NaCl + NNS	208	1.55	^b^MART	32 °C; 73%	Sprint duration	↑ (0.19)
Devlin et al. (2001a) [71]	7 M, sub-elite cricketers	56 ± 6	EX; 28 °C; 60 min	0	2.8	Water	60	0.80	^c^MMRT	16 °C; 60%	20-m shuttle runs	↑ (0.30)
Cheuvont et al. (2006) [51]	8 M, physically active	52 ± 6	HT	Testing at 0, 30 and 60	2.7	Water	185–240	1.00	15 s WAnT	22 °C; 65%	Abs. mean PO; Rel. mean PO; Abs. PPO; Rel. PPO; rate of fatigue	No effect
Edwards et al. (2007a) [58]	11 M, moderately active soccer players	50.9 ± 4.0	EX; 19-25 °C; 90 min	0	2.4	Water	90	0.80	^d^Yo-Yo Test	NS	Distance covered	↑ (ES unknown)
Maxwell et al. (2009a) [22]	8 M, unacclimated, game players	59.9 ± 8.0	EX (H/M); 36 °C; 90 min	>12 h	3.9	Water	>12 h	1.50	^e^IST	36 °C; 49%	TW; Abs. PPO; Rel. PPO (during the IST, RSB 1 and RSB 2)	TW (0.97) and Abs. PPO (0.79) ↑ on RSB 2 only.
Maxwell et al. (2009b) [22]	8 M, unacclimated, game players	59.9 ± 8.0	EX (H/M); 36 °C; 90 min	>12 h	3.9	Water	>12 h	1.00	^e^IST	36 °C; 49%	TW; Abs. PPO; Rel. PPO (during the IST, RSB 1 and RSB 2)	No effect
Kraft et al. (2011) [75]	10 M	NS	WI	45	3.0	Water	158–178	1.00	^f^IST	NS	Abs. mean PO; Abs. PPO; s >90 rpm; Rate of fatigue	No effect
Owen et al. (2013a) [59]	13 M, semi-professional soccer players	54 ± 3	EX; 19 °C; 105 min	5	2.5	Water	110	0.89	^d^Yo-Yo Test	19 °C; 59%	Distance covered	No effect
Owen et al. (2013b) [59]	13 M, semi-professional soccer players	54 ± 3	EX; 19 °C; 105 min	5	2.5	Water	110	0.51	^d^Yo-Yo Test	19 °C; 59%	Distance covered	No effect

Exercise intensity is described as high (H), vigorous (V) or moderate (M), in accordance with classifications outlined by Norton et al. [86]. Values are Hedges’ *g* effect sizes

*Abs*. absolute, *Dh* dehydration, *ES* effect size, *EX* exercise, *HT* heat, *IST* intermittent sprint test, *MART* maximal anaerobic running test, *Max*. maximum, *MMRT* maximal multistage running test, *NNS* non-nutritive sweetener, *NS* not specified, *PO* power output, *PPO* peak power output, *RH* relative humidity, *Rel*. relative, *Total REC* time from completing the dehydration protocol to commencing the subsequent task, *TW* total work, *WAnT* Wingate Anaerobic Test, *WI* warm water immersion

^a^The intermittent sprint test (IST) comprised of five 5-s sprints at 3-min intervals on a cycle ergometer

^b^The maximal anaerobic running test (MART) involved repeated 20-s runs on a treadmill, at increasing intensities, with 100-s passive recovery between runs until volitional exhaustion

^c^The maximal multistage running test (MMRT) involved repeated 20-m runs between two points, at increasing intensity

^d^The Yo-Yo intermittent recovery test is a soccer-specific performance test that comprises of 20-m shuttle runs separated by 10 s jog recovery. Running speed during the test is incremental, and maximal performance is indicated by total distance covered

^e^The IST comprised of a 36-min repeated sprint exercise divided into 2-min periods of a 4-s sprint and 100 s of active recovery (35% VO2 _max_) and 16 s passive rest. A repeated sprint bout (RSB) involving 5 × 2 s sprints with 18 s active recovery was also completed after the 8th and 16th sprints (RSB 1 and RSB 2). All testing was completed on a cycle ergometer

^f^The IST comprised of a 3-min warm up followed by 6 × 15 s maximal sprints separated by 30 s active recovery on a cycle ergometer

**Table 3:** Characteristics of research studies evaluating athletic performance on resistance exercise tasks

Citation	Participants	Dh protocol; ambient temperature; duration	Total REC (min)	Dh trial BM loss (%)	Fluid type	Fluid assimilation time (min)	Fluid intake (L kg BM lost^−1^)	Resistance exercise task	Performance outcomes(s)	Performance outcomes significantly affected (Hedges’ *g*)
Montain et al. (1998) [66]	8 M, physically active	EX (M); 40 °C; 2–3 h	Testing from 3–8 h	4.0	Water	3–8 h	NS	Knee extension	ET >50% MVC; MVC pre-ET test, 30-s post-ET test and >30-s post-ET test	ET >50% MVC ↑ (2.70) MVC ↓ (ES unknown)
Greiwe et al. (1998) [52]	7 M, unacclimated	HT	120	3.8	Water	306	1.00	Knee extension and elbow flexion	Peak torque; ET 100% MVC	No effect
Bigard et al. (2001) [53]	11 M, unacclimated, physically active	HT	180	3.0	NaCl + water	120	1.00	Knee extension	MVC, ET 25% MVC; ET 75% MVC	ET 25% MVC ↑ (0.22)
Schoffstall et al. (2001) [54]	10 M, competitive power lifters	HT	120	1.7	Water	120	1.10	Bench Press	1 RM	↑ (0.24)
Del Coso et al. (2008) [46]	7 (NS), acclimated, endurance cyclists	EX (V); 36 °C; 2 h	0	3.7	Mineral water	120	0.90	Knee extension	MVC	No effect
Kraft et al. (2010) [74]	10 M, recreationally strength trained	WI	≥45	3.1	Water	165	1.00	^g^Full body resistance exercise protocol	Repetitions completed at 12 RM	↑ (0.87)
Ali et al. (2013) [57]	10 M, university-level soccer players	EX (V); 22 °C; 90 min	0	2.9	Water	90	0.50	MVC _(3.14 and 1.05 rad/s)_ knee flexion and extension	Peak torque; TW; Abs. mean PO	No effect
								Knee extension and elbow flexion	Peak torque; mean torque	No effect
Wilson et al. (2014) [60]	8 M, licenced jockeys	EX; 20 °C; 45 min	0	1.8	Water	~35	1.00	Chest-press and knee flexion	Max. strength	↑ Chest (5.57) and leg (1.05) max. strength
Rodrigues et al. (2014) [49]	10 M, unacclimated, physically active	EX (V); 37 °C; 91 min	30	2.0	Water	121	NS	Knee extension and elbow flexion	Peak torque	Knee extensor peak torque ↑ (0.85)

Exercise intensity is described as high (H), vigorous (V) or moderate (M), in accordance with classifications outlined by Norton et al. [86]. Values are Hedges’ *g* effect sizes

*Abs*. absolute, *Dh* dehydration, *ES* effect size, *ET* endurance time, *EX* exercise, *HT* heat, *MVC* maximal voluntary contraction, *NS* not specified, *PO* power output, *RH* relative humidity, *RM* repetition maximum, *Total REC* time from completing the dehydration protocol to commencing the subsequent task, *TW* total work, *WI* warm water immersion

^g^The full body resistance exercise protocol measured total repetitions in three sets of bench press, lat pull down, overhead press, barbell curl, triceps and leg press exercise at 12 RM

**Table 4:** Characteristics of research studies evaluating athletic performance on sport-specific exercise tasks

Citation	Participants	VO_2 max_	Dh protocol; ambient temperature; duration	Total REC (min)	Dh trial BM loss (%)	Fluid type	Fluid assimilation time (min)	Fluid intake (L∙kg BM lost^−1^)	Sport-specific exercise task	Performance outcomes(s)	Performance outcomes significantly affected (Hedges’ *g*)
Devlin et al. (2001b) [71]	7 M, sub-elite cricketers	56 ± 6	EX; 28 °C; 60 min	0	2.8	Water	60	0.80	Cricket bowling	Accuracy (line and length); velocity	↑ Bowling accuracy for line (0.85) and length (0.85)
Ali et al. (2011) [35]	10 (0 M), soccer players	47 ± 4	EX; 17 °C; 90 min	0	1.4	Water	90	1.07	^h^LSPT	Movement time; penalty time; performance time	No effect
Fritz et al. (2013) [72]	13 M, elite squash players	NS	EX	NS	1.3	Water	NS	0.40	^i^Ghosting Test	TT	↓ (0.55)
Owen et al. (2013c) [59]	13 M, semi-professional soccer players	54 ± 3	EX; 19 °C; 105 min	5	2.5	Water	110	0.89	^h^LSPT	Movement time; penalty time; performance time	No effect
									^j^LSST	Time taken; shot speed; points per shot	No effect
Owen et al. (2013d) [59]	13 M, semi-professional soccer players	54 ± 3	EX; 19 °C; 105 min	5	2.5	Water	110	0.51	^h^LSPT	Movement time; penalty time; performance time	No effect
									^j^LSST	Time taken; shot speed; points per shot	No effect
Wilson et al. (2014) [60]	8 M, licenced jockeys	NS	EX; 20 °C; 45 min	0	1.8	Water	~35	1.00	Simulated race ride	Pushing frequency	↑ (1.46)
									Go-No-Go task	SRT	No effect

Values are Hedges’ *g* effect sizes

*Dh* dehydration, *EX* exercise, *LIST* Loughborough Intermittent Sprinting Test, *LSPT* Loughborough Soccer Passing Test, *LSST* Loughborough Shooting Test, *Max*. maximum, *NS* not specified, *RH* relative humidity, *SRT* simple reaction time, *Total REC* time from completing the dehydration protocol to commencing the subsequent task, *TT* time trial

**Table 5:** Characteristics of research studies evaluating athletic performance on balance exercise tasks

Citation	Participants	Dh protocol; ambient temperature; duration	Total REC (min)	Dh trial BM loss (%)	Fluid type	Fluid assimilation time (min)	Fluid intake (L kg BM lost^−1^)	Balance exercise task	Performance outcomes(s)	Performance outcomes significantly affected (Hedges’ *g*)
Erkmen et al. (2010) [61]	17 M, physically active	EX (V); 21–24 °C; 60 min	Testing at 0 and 20 min	3.3	Water	60/80	1.00	One-leg stand static balance test	Eyes close and eyes open ^k^OSI (0 min post-Dh) Eyes close and eyes open ^k^OSI (20 min post-Dh)	↓ Eyes open OSI 0 min post-Dh (1.12)
Ely et al. (2012a) [67]	32 M, unacclimated	EX; 50 °C; 3 h work/rest	90	4.1	NaCl + water	270	1.00	20-s dynamic balance test	^k^OSI; ^l^ mean deflection; time spent stable (Testing at 10 °C, 20 °C, 30 °C and 40 °C)	No effect

Exercise intensity is described as high (H), vigorous (V) or moderate (M), in accordance with classifications outlined by Norton et al. [86]. Values are Hedges’ *g* effect sizes

*Dh* dehydration, *EX* exercise, *OSI* overall stability index, *Total REC* time from completing the dehydration protocol to commencing the subsequent task

^h^During the Loughborough Soccer Passing Test (LSPT), participants completed a random sequence of eight short and long passes of a soccer ball towards a target, as quickly as possible with the fewest time penalties

^i^The ‘Ghosting Test’ is a squash-specific movement test. Participants were instructed to collect a half-ball that was placed on three racquets positioned around the court, move to the ‘T’, and then to the next racquet at the opposite corner as quickly as possible

^j^During the In the Loughborough Shooting Test (LSST), participants were required to sprint ~12 m, then pass, control and shoot the ball at targets within the goal area

^k^The overall stability index (OSI) is an indicator of a subject’s ability to balance on a platform. A higher OSI indicates poorer balance performance

^l^Mean deflection was defined as the average position of the subject during the balance test. A higher mean deflection indicates more displacement and poorer balance performance

**Table 6:** Characteristics of research studies evaluating cognitive performance

Citation	Participants	Dh protocol; ambient temperature; duration	Total REC (min)	Dh trial BM loss (%)	Fluid type	Fluid assimilation time (min)	Fluid intake (L∙kg BM lost^−1^)	Cognitive domains assessed	Ambient temperature; RH; airflow	Cognitive domains significantly affected by fluid intake (Hedges’ *g*)
Cian et al. (2001a) [55]	7 M, unacclimated, endurance trained	EX (V); 25 °C; 120 min	Testing at 140 and 240 min	2.7	CHO + electrolyte	80/180	1.00	Memory Perceptive discrimination Psychomotor function/processing speed Mood	NS	↑ Memory 2-h post-Dh
Cian et al. (2001b) [55]	7 M, unacclimated, endurance trained	HT	Testing at 140 and 240 min	2.6	CHO + electrolyte	80/180	1.00	Memory Perceptive discrimination Psychomotor function/processing speed Mood	NS	↑ Memory 2-h post-Dh ↓ Fatigue
Grego et al. (2004) [48]	8 M, endurance trained cyclists	EX (V); 20–21 °C; 180 min	5	4.1	Water	185	0.73	Perceptual discrimination Memory/processing speed	NS	No effect
Serwah et al. (2006a) [68]	8 M	EX (V); 31 °C; 90 min	3	1.7	Water	9	1.00	Psychomotor function/processing speed	NS	No effect
Serwah et al. (2006b) [68]	8 M	EX (V); 31 °C; 90 min	3	1.7	Water	90	0.50	Psychomotor function/processing speed	NS	No effect
Edwards et al. (2007b) [58]	11 M, moderately active soccer players	EX; 19–25 °C; 90 min	0	2.4	Water	90	0.80	Visual scanning/processing speed	NS	No effect
Adam et al. (2008a) [56]	8 (6 M), physically active soldiers	HT	120	3.0	NS	300	NS	Psychomotor function/processing speed Psychomotor function Perceptive discrimination Visual scanning/vigilance	20 °C; 50%; 1 m/s	No effect
Adam et al. (2008b) [56]	8 (6 M), physically active soldiers	HT	120	3.0	NS	300	NS	Psychomotor function/processing speed Psychomotor function Perceptive discrimination Visual scanning/vigilance	2 °C; 50%; 2.2 m/s	No effect
D’Anci et al. (2009a) [73]	16 M, university athletes	EX; 60 min	NS	2.0	Water	60	NS	Memory Psychomotor function/processing speed Arithmetic/processing speed Visual scanning/vigilance Spatial processing Mood	NS	↑ Psychomotor function/processing speed ↓ Anger, fatigue, depression, tension and confusion; ↑ vigour
D’Anci et al. (2009b) [73]	13 (0 M), university athletes	EX; 60–75 min	NS	1.7	Water	60-75	NS	Memory Psychomotor function/processing speed Arithmetic/processing speed Visual scanning/vigilance Spatial processing Mood	NS	↑ Psychomotor function/processing speed ↓ Anger, fatigue, depression, tension and confusion; ↑ vigour
Ganio et al. (2011) [69]	24 M, physically fit	EX; 28 °C; 40 min	20	1.6	Water	60	NS	Psychomotor function/processing speed Visual scanning/vigilance Memory/processing speed Learning/memory Logical reasoning/processing speed Mood	23 °C	↑ Psychomotor function/processing speed and memory/processing speed ↓ Fatigue and tension
Ely et al. (2012b) [67]	32 M, unacclimated	EX; 50 °C; 3 h W/R	90	4.1	NaCl + water	270	1.00	Psychomotor function/processing speed Memory/processing speed Logical reasoning/processing speed Mood	Testing at 10, 20, 30 and 40 °C	No effect
Wilson et al. (2014) [60]	8 M, licenced jockeys	EX; 20 °C; 45 min	0	1.8	Water	~35	1.00	Response inhibition	NS	No effect
Wittbrodt et al. (2015a) [70]	12 M, recreationally active	EX (V); 32 °C; 50 min	NS	1.5	Water	>50	1.00	Psychomotor function/processing speed Memory Perceptive discrimination Visual scanning/processing speed	32 °C; 65%	No effect
Wittbrodt et al. (2015b) [70]	12 M, recreationally active	EX (V); 32 °C; 50 min	NS	1.5	Water	>50	0.80	Psychomotor function/processing speed Memory Perceptive discrimination Visual scanning/processing speed	32 °C; 65%	No effect

Exercise intensity is described as high (H), vigorous (V) or moderate (M), in accordance with classifications outlined by Norton et al. [86]. Values are Hedges’ *g* effect sizes

*CHO* carbohydrate, *Dh* dehydration, *EX* exercise, *HT* heat, *NS* not specified, *Total REC* time from completing the dehydration protocol to commencing the subsequent task, *W*/*R* work rest cycle

### Athletic Performance

Forty-nine trials (*n* = 461, 95% male, excluding Del Coso et al. [46] where gender was NS) examined the effect of fluid intake on athletic performance tasks. Findings from research studies that evaluated athletic performance are summarized in Tables 1, 2, 3, 4 and 5. (Full details are presented in Additional file 1: Table S2, S3, S4, S5 and S6).

#### Continuous Exercise Performance

Twenty-two trials (*n* = 170 subjects, 98% male) measured the effect of fluid intake on continuous exercise performance (Table 1). The majority of testing was completed on well-trained individuals (mean VO_2 max_ 57.5–68.4 mL kg^−1^ min^−1^) [24, 25, 29, 33, 36, 47, 50, 62, 63, 76, 77] (*n* = 13 trials). In *n* = 11 trials, exercise was performed in a warm or hot environment (30–40 °C), by acclimated (*n* = 2) [63, 77] and unacclimated (*n* = 5) [29, 36, 47, 62, 76] subjects, where environmental adaptation was specified. The remaining trials were completed under thermoneutral (18–25 °C) [24, 25, 29, 37, 41, 42] (*n* = 7) or cold (2–10 °C) [41, 42] (*n* = 2) conditions, where ambient temperature was specified. Fluid intake significantly improved continuous exercise performance in 13 out of the 22 trials reviewed.

#### Meta-analyses and Meta-regression Analyses

Eighteen trials (*n* = 139 subjects, 97% male) were included in the meta-analysis examining the effect of fluid consumption on continuous exercise performance. Four continuous exercise trials included in the systematic review were omitted from the meta-analysis on the basis of: (1) duration of the TTE performance test was capped (*n* = 2) [36, 62]; (2) Rosendal score <50% (*n* = 1) [47] and (3) extreme outlier, exceeding the mean effect estimate by >3 SD with a studentized residual of 2.82 (*n* = 1) [64], with the results possibly confounded by fatigue. In this study [64], untrained participants completed 1-h dehydrating exercise at 32 °C before commencing a TTE test at 80% VO_2 max_, without any recovery. All other investigations completed on participants with a VO_2 max_ less than 50.0 mL kg^−1^ min^−1^, i.e. untrained or physically active, employed passive methods of dehydration or allocated ~2 h recovery post-active dehydration [41, 42]. Excluding this trial did not influence the results of the meta-analysis (Hedges’ *g* = 0.48, 95% CI 0.33, 0.63), thus it was removed.

**Fig. 3:**
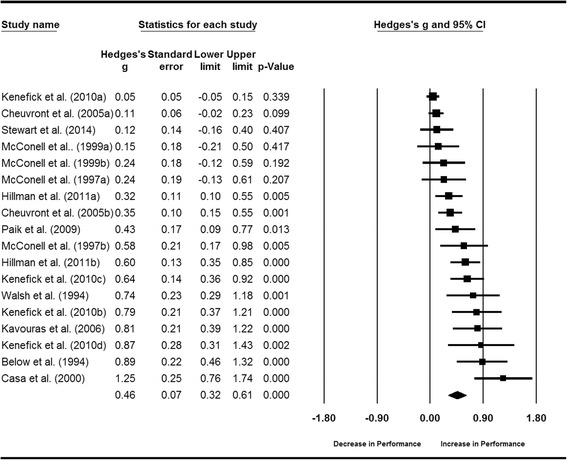
Forest plot displaying the effect of fluid intake on continuous exercise performance. Size of the squares is proportional to the weight of the study

The weighted mean treatment effect summary indicates fluid intake following a period of dehydration significantly improved continuous exercise performance (*g* = 0.46, 95% CI 0.32, 0.61) (Fig. 3). Data were normally distributed (Shapiro-Wilk Test, *p* > 0.05). High heterogeneity was evident between trials (*I* ^2^ = 80.5, *p* < 0.01). Subsequent analyses (see below) determined that 82% of variation between trials is due to differences in the ambient environmental temperature at which the exercise was performed. Thus, sensitivity analysis was completed with trials sub-grouped by environmental temperature, where cold-thermoneutral = ≤25 °C and warm-hot = >25 °C. The magnitude and statistical significance of the treatment effect was stable during sensitivity analysis where trials were sequentially removed, with Hedges’ *g* ranging between 0.29–0.35 and 0.70–0.81 for cold-thermoneutral and warm-hot ambient temperature subgroups, respectively (all *p*s <0.01). Findings are comparable across different levels of correlation (*R* = 0.50, 0.74, 0.84 and 0.94), therefore the meta-analysis (and subsequent meta-regression analyses) are robust to the imputed *R* = 0.84 (full details of sensitivity analyses are presented in Additional file 1: Table S8 and S9).

**Fig. 4:**
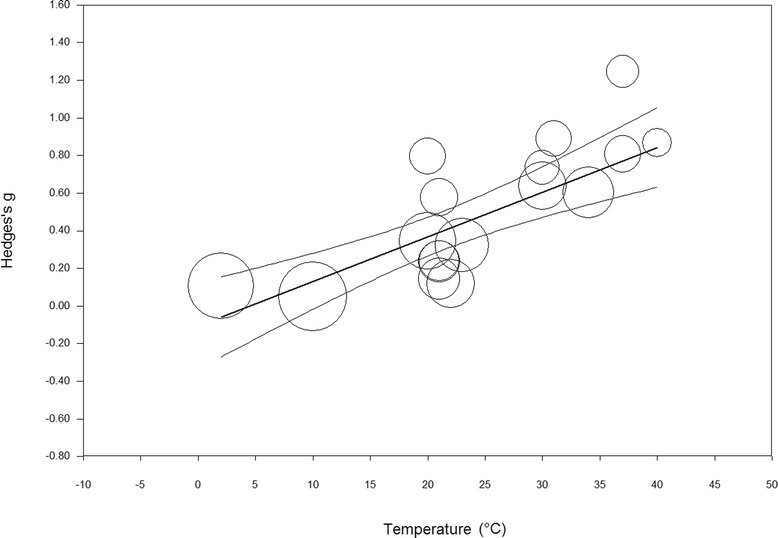
Correlation between change in ambient environmental temperature and change in continuous exercise performance (Hedges’ *g*). *Circle diameter* corresponds to the weight of each study. Hedges *g* = −0.105 + 0.024 × temperature (°C)

One continuous exercise trial [50] was excluded from the simple meta-regression analysis to determine the relationship between changes in ambient temperature and the magnitude of the weighted mean effect after failing to report environmental temperature at the time of exercise performance. Analyses of the remaining 17 trials (*n* = 129 subjects, 97% male) detected a strong significant correlation (*R* ^2^ = 0.82, *p* < 0.01) between these parameters (Fig. 4). Therefore, fluid intake may enhance continuous exercise performance to a greater extent at increasing environmental temperatures.

**Fig. 5:**
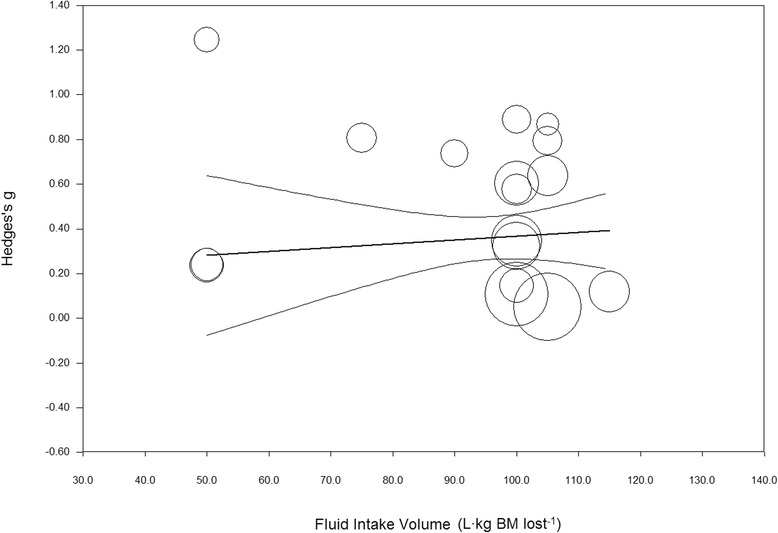
Correlation between change in fluid intake (L kg BM lost^−1^) and change in continuous exercise performance (Hedges’ *g*) controlling for ambient environmental temperature, exercise duration, level of dehydration and the ecological validity of the exercise protocol. *Circle diameter* corresponds to the weight of each study. Hedges *g* = −0.557 + 0.002 × fluid volume (L kg BM lost^−1^) + 0.025 × temperature (°C) + 0.011 × exercise duration (min) + 0.218, if ecologically valid + 0.0126 × level of dehydration (% BM lost)

The influence of environmental temperature, exercise duration, ecological validity of the exercise protocol and the level of dehydration were controlled in the modelling of the relationship between the volume of fluid consumed (L kg BM lost^−1^) and the magnitude of the weighted mean effect (Fig. 5). The volume of fluid administered ranged between 0.50–1.15 L kg BM lost^−1^. No correlation was observed between these parameters (*p* = 0.625) (Table 7). Mean exercise duration ranged between 4 and 30 min, since no trials involving an exercise task lasting >30 min were eligible for inclusion (as outlined above). There was a trend for fluid intake to improve performance to a greater extent with increasing exercise duration (*p* = 0.071). The majority of trials (*n* = 12) measured continuous exercise performance on an ecologically valid exercise protocol, e.g. total work or power output completed within a predefine timeframe (*n* = 10) [25, 29, 41, 42] or time to complete a set distance (*n* = 2) [37, 63]. The remaining trials (*n* = 5) employed a fixed-power TTE exercise protocol with low ecological validity [24, 33, 76, 77]. No significant correlation was observed between the ecological validity of exercise protocol employed and the magnitude of the weighted mean effect (*p* = 0.188), or the level of dehydration and the magnitude of the weighted mean effect (*p* = 0.845).

**Table 7:** Summary of moderator variables for the meta-regression analysis of the effect of fluid volume on the magnitude of the weighted mean treatment effect

Covariate	Coefficient (95% CI)	*p* value	*R* ^2^
Fluid volume	0.002 (−0.006, 0.009)	0.625	0.91
Temperature	0.025 (0.015, 0.036)	<0.001	
Exercise duration	0.011 (−0.001, 0.023)	0.071	
Ecological validity	0.218 (−0.124, 0.561)	0.188	
Level of dehydration	0.013 (−0.126, 0.151)	0.845	

One trial [76] failed to report time from commencing fluid ingestion to beginning the subsequent performance task and was excluded from the multiple regression analysis of fluid assimilation time vs. Hedges’ *g*. Modelling of this relationship corrected for the influence of environmental temperature and type of exercise protocol. Exercise duration was omitted from the model due to collinearity with fluid assimilation time (VIF = 3.18, where all other analyses yielded values ≤1.7). Fluid assimilation time ranged between 45 and 390 min. Analyses of the 16 eligible trials (*n* = 121 subjects, 94% male) did not detect a significant influence of fluid assimilation time on the weighted mean effect (*p* = 0.11).

#### Intermittent Exercise

Ten trials (*n* = 95 male subjects) evaluated intermittent exercise performance (Table 2). Exercise was undertaken in hot (32–36 °C) [22, 65], thermoneutral (19–22 °C) [51, 59] and cold (16 °C) [71] environmental conditions, where ambient temperature was specified. The majority of testing was done using team sport participants (i.e. individuals who are accustomed to intermittent exercise) [22, 58, 59, 71]. Participants in the remaining trials were untrained [65], physically active [51] or endurance cyclists [33], where the participant population was defined. Fluid intake (0.8–1.55 L kg BM lost^−1^) significantly improved intermittent exercise performance on 4 out of the 10 trials [22, 58, 65, 71]. The magnitude of improvement ranged from small to large (Hedges *g* = 0.19–0.97).

#### Resistance Exercise

Nine trials (*n* = 83 subjects, 100% male, excluding Del Coso et al. [46] where gender was NS) evaluated resistance exercise performance (Table 3). Across the 8 trials reviewed, 22 separate performance tests were identified. The majority were knee extension or elbow flexion exercise tasks, at variable intensities (*n* = 18 tasks) [46, 49, 52, 53, 57, 66], although 2 trials measured performance via repetition lifts [54, 74]. Individuals who were accustomed to performing resistance exercise were rarely studied [54, 74]. Fluid intake (1.0–1.10 L kg BM lost^−1^) significantly improved performance on 7 of 22 resistance exercise tasks completed across 5 trials (Hedges’ *g =* 0.22–5.57) [49, 53, 54, 60, 66, 75], and significantly decreased performance on 1 task [66].

#### Sport-Specific Exercise

Six trials (*n* = 64 subjects, 84% male) evaluated athletic performance on exercise tasks that were specific to either cricket (*n* = 1) [71], soccer (*n* = 3) [35, 59], squash (*n* = 1) [72] or racehorse riding (*n* = 1) [60] (Table 4). All participants were experienced on the sporting activity for which they were assessed. Fluid intake had no effect on soccer players’ ball-skills (e.g. passing and shooting) [59]. However, squash-specific movements, cricket bowling accuracy and racehorse riding demonstrated moderate to large performance improvements with fluid intake (0.4–1.0 L kg BM lost^−1^).

#### Balance Exercise

Two trials (*n* = 49 male subjects) examined balance performance (Table 5). A significant positive effect of fluid intake was documented for 1 out of 8 balance-related tests completed across both trials.

### Cognitive Performance and Mood State Outcomes

Fifteen trials (*n* = 182 subjects, 90% male) examined the effect of fluid intake on cognitive performance and/or mood. Major findings are summarized in Table 6. (Full details are presented in Additional file 1: Table S7). Across the 15 trials reviewed, 49 neuropsychological tests were identified. Evidence indicating a beneficial effect of fluid intake on cognitive performance was observed on 5 cognitive tests completed across 5 trials [55, 69, 73]. Cognitive domains affected were memory, psychomotor function and processing speed. Four out of the 6 trials evaluating the influence of fluid intake on mood state observed significant positive effects [55, 69, 73], as indicated by decreased subjective ratings of fatigue, anger, depression, tension and confusion and increased vigour.

## Discussion

Individuals prone to dehydration (e.g. athletes and manual workers) may have limited opportunity to adequately rehydrate prior to performing physically or cognitively demanding activities. The present systematic review and meta-analysis examines evidence for the effects of fluid intake on subsequent athletic and cognitive performance following dehydrating sweat loss. A beneficial effect for fluid intake was strongest when athletic performance involved continuous exercise tasks. Further, the magnitude of improvement appeared greater when the continuous exercise was performed at elevated environmental temperatures and over longer exercise durations. Whilst the volume of fluid consumed (relative to BM lost) did not appear to influence the size of the treatment effect, fluid intake at levels complying with current recommendations for completely replacing lost fluid (1.25–1.50 L kg BM lost^−1^) [16, 17] are yet to be thoroughly investigated. Evidence for a beneficial effect of fluid intake on intermittent, resistance and sport-specific exercise performance and cognitive function or mood is less apparent and requires further elucidation.

The weighted mean effect suggests that fluid ingestion following a period of dehydration significantly improves continuous exercise performance, compared to control conditions (no fluid or negligible fluid intake). Individual estimates all indicated a beneficial effect from fluid intake; however, the magnitude of the improvement was heterogeneous (*I* ^2^ = 80.5%) which may reflect differences in the methodologies employed between studies. Simple meta-regression determined that 82% of variation between trials can be attributed to differences in the ambient environmental temperature at which subsequent exercise was performed, with fluid intake demonstrating greater efficacy under heat stress conditions. The decline in aerobic performance that occurs with hypohydration has largely been attributed to circulatory strain, whereby reductions in blood volume limit oxygen transport to the exercising muscle [78, 79]. Under elevated environmental temperatures, blood flow is also redirected to the skin facilitating evaporative cooling, augmenting circulatory strain and further impairing exercise performance [41]. These physiological perturbations are typically characterized by increased heart rate and core temperature [41, 78, 79]. Hence, thermoregulatory parameters were monitored in many of the studies reviewed (11/15) [24, 25, 29, 33, 36, 37, 41, 42, 47, 62, 63, 76, 77]. The majority of reviewed studies reported that consumption of fluid was associated with significant reductions in core or rectal temperature (7/11) [36, 41, 42, 47, 62, 63, 76] and heart rate (6/10) [36, 41, 42, 47, 62, 63, 76, 77] at various time points during continuous exercise performance (i.e. for at least one fluid intervention). Thus, fluid intake may offset circulatory strain typically observed when exercise is undertaken in warm environments. The multiple meta-regression analysis also suggests that differences in the duration of the continuous exercise performed may account for a proportion of the heterogeneity observed between experimental trials, with exercise performed over longer durations yielding greater benefit from fluid intake than short duration exercise. However, as the majority of performance tests included in the analysis were relatively short in duration (4–30 min), we cannot be certain that this relationship would hold true over longer exercise durations (i.e. 2–8 h).

Results of the meta-regression failed to indicate a statistically significant relationship between the volume of fluid consumed and continuous exercise performance improvements. However, the majority of trials tested a quantity of fluid that was within a narrow fluid intake range (i.e. 1.0–1.05 L kg BM lost^−1^, *n* = 13 out of 18). Hence, the performance effects associated with ingesting a comparably small volume of fluid (e.g. ≤0.75 L kg BM lost^−1^) or an amount consistent with recommended guidelines (e.g. 1.25–1.50 L kg BM lost^−1^) remains uncertain. Three experimental investigations have examined the dose-response effect of ingested fluid volume on continuous exercise performance following a period of dehydration with the results demonstrating inconsistent findings [21, 24, 25]. Unfortunately, the investigation with the greatest contrast in fluid volumes (i.e. 0.75 vs. 1.50 L kg BM lost^−1^ [21]) did not employ a ‘no fluid’ control and was unable to be included in the meta-analysis. Findings from previous studies suggest that fluid intake during exercise exceeding that dictated by thirst may not provide additional performance benefits [80]. However, only three of the publications reviewed measured subjective thirst within the investigation (and these studies did not test different fluid volumes, i.e. only one intervention vs. control). Therefore, it is not clear whether the equivocal effect of fluid intake volume can be attributed to thirst sensation. Based on current evidence, prescribing fluid volumes required to optimize performance on a subsequent continuous exercise task requires clarification.

If relatively small and large fluid intakes elicit comparable treatment effects, individuals who have limited time to rehydrate prior to performing aerobic activities may opt to consume smaller fluid boluses, delaying complete rehydration until circumstances permit (e.g. overnight). This strategy may reduce the probability of the drinker experiencing volume-induced GI discomfort during subsequent activity, which may occur when larger fluid volumes are ingested [25]. Only one of the 42 publications reviewed monitored GI symptomology [25]. In this study, subjective ratings of GI discomfort following different fluid intakes (~0.5 vs. 1.0 L kg BM lost^−1^) were described as mild to moderate and moderate to high on each trial, respectively. This suggests that larger fluid volumes are likely to induce some degree of participant discomfort which may compromise performance. However, research examining continuous exercise performance following two volumes of fluid intake (i.e. 0.75 vs. 1.50 L kg BM lost^−1^) demonstrated significantly faster (~3.0%) running performance associated with the larger bolus [21]. Importantly, this study employed a prolonged (i.e. overnight) rehydration period reducing the probability of severe GI disturbance and allowing ingested fluid to equilibrate throughout the body. Further research examining exercise performance (and GI symptoms) when large fluid volumes are ingested over short rehydration periods is warranted.

The effect of fluid intake on intermittent, resistance, sport-specific and balance exercise types remains unclear. It appears that fluid ingestion following a period of dehydration may improve performance on subsequent intermittent, resistance and sport-specific exercise tasks. However, methodological differences make comparison of results across trials challenging.

In regards to intermittent exercise, 4 of 10 trials (*n* = 95 male subjects) observed a significant positive effect of fluid intake on performance, whilst no trial reported a significant performance decrement. Similarly to the results from continuous exercise, beneficial effects of fluid intake are apparent when intermittent exercise tasks have been completed in warm environments [22, 65]. The impact of task duration may also influence the likelihood of observing performance effects, with longer duration tasks more regularly demonstrating a performance enhancement associated with fluid ingestion [22, 65]. For instance, Maxwell et al. [22] observed that fluid intake only benefited performance on a second repeated sprint bout completed in the latter stages of testing.

Concerning resistance exercise, 6 of 9 trials (*n* = 83 subjects, 93% male) observed a significant positive effect of fluid intake on performance. One trial reported that fluid intake was detrimental to performance [66]. However, results from this study need to be interpreted with caution as the strength performance task was performed following an endurance task that varied in duration. Evidence indicating a beneficial effect of fluid intake on resistance exercise performance appears stronger when tests of muscular endurance, rather than tests of muscular strength, are employed [53]. Findings from this systematic review demonstrate significantly improved performance on 3 out of the 4 submaximal intensity resistance exercise tasks [53, 66, 74]. In contrast, performance on only 4 out of 15 maximal intensity tests demonstrated improvement with fluid intake [49, 54, 60]. Current evidence is inadequate to determine the influence of other variables (e.g. participant population, mode of dehydration) on the effect of fluid intake. Further research examining the effects of fluid intake on resistance exercise performance using standardized procedures is required.

The 6 trials (*n* = 64 subjects, 84% male) that evaluated the effect of fluid intake on sport-specific exercise performance exhibited considerable heterogeneity, with tests of cricket [71], soccer [35, 58, 59], squash [72] and racehorse riding [60] performance all being employed. Whilst the majority of sports-specific research has demonstrated no impact of fluid consumption on subsequent performance, the paucity of data and lack of replication studies makes it difficult to determine an overall effect of fluid intake on sport-specific exercise performance.

The present systematic review identified 14 trials (*n* = 174 subjects, 90% male) examining the effect of fluid intake following a period of dehydration on cognitive function and mood state. Evidence indicating a beneficial effect of fluid intake on cognitive performance was only observed in some studies [55, 73], and there was no clear indication of greater treatment efficacy on a particular cognitive domain. However, some limitations to the current evidence exist. In 4 trials, the cognitive assessment was conducted ≤5 min after concluding the dehydration protocol [48, 58, 68]; further 4 trials did not provide the necessary information to calculate the amount of time between the conclusion of the dehydration protocol and commencement of the cognitive tests [58, 70, 73]. Prior research indicates that acute exercise has a small positive effect on cognitive performance (typically dissipating within ~15 min of exercise cessation) [81], whilst elevated core temperature via heat stress may provide additional cognitive burden [82]. Therefore, the residual effects of physiological stressors used to induce dehydration in these trials may obscure any influence of fluid intake on cognitive performance. Investigations examining the effect of hydration on cognitive performance should also employ neuropsychological tests that have previously demonstrated sensitivity to nutritional interventions [34, 83, 84]. Yet, only two studies included in the present review selected cognitive tests on this basis [56, 73]. The majority did not provide any rationale for their chosen method of assessment [48, 55, 58, 67, 68, 70], increasing the likelihood of false-negative reports. Fluid consumption positively influenced mood state (measured as reduced anger, fatigue, depression, tension and confusion) in 4 out of the 6 trials where it was measured [55, 69, 73]. Whilst this may suggest that self-reported mood state questionnaires are more sensitive to the effects of fluid intake than objective tests of cognitive function, subjective mood ratings were only influenced by fluid intake during trials where significant cognitive effects were also observed, i.e. effects on mood and cognition were not independent of one another. Collectively, it appears that the influence of fluid intake on mood and cognitive performance is still poorly understood and requires further research employing tasks with demonstrated sensitivity.

This review does contain a number of limitations. Firstly, only studies with accessible full text articles written in English were included. Second, three of the studies reviewed [69, 71, 77] examined rehydration in combination with another placebo treatment (studies were excluded if fluids were co-administered with another experimental treatment). Thus, participants’ perceptions regarding the expected treatment may have influenced these results. Third, as oral fluid replacement cannot be blinded, it is conceivable that the placebo effect may account for a small amount of benefit observed with rehydration. However, it was necessary to exclude research studies that blinded participants to hydration status using intravenous methods because the infusion does not accurately mimic the physiological effects of oral rehydration. Fourth, the present review elected to compare against a “no fluid” or “negligible fluid” control condition, because a euhydrated control may be confounded by the effects of the dehydration protocol itself (i.e. hyperthermia or fatigue). However, using this comparison, we cannot determine whether fluid intake fully or partially restored performance to euhydrated levels. Similarly, fluid ingestion may have minimal or no effect on athletic or cognitive performance if the outcome measured is not sensitive to the effects of modest fluid losses. Fifth, where fluid was administered at the time of dehydration (i.e. *concurrent* fluid intake), rather than following dehydration (i.e. *subsequent* fluid intake), different physiologic responses to the dehydration protocol may occur on control and intervention trials, e.g. decreased core temperature leading to reduced central fatigue. This could have implications for subsequent athletic performance, and consequently, the magnitude of the overall treatment effect. Sixth, fluid intake ≤200 mL was considered ‘negligible’ and included within the definition of control conditions. However, one study has reported that ingesting 100 mL of fluid (25 mL boluses at 5-min intervals during exercise) increased TTE following exercise-induced dehydration [85]. Thus, trials administering ≤200 mL fluid to dehydrated control subjects may underestimate the true magnitude of the treatment effect.

## Conclusions

Collectively, the results of the present review suggest that individuals who have limited opportunity to adequately rehydrate prior to performing continuous exercise in a heated environment should consume fluid, even if the body water deficit is modest (1.3% reduction in BM) and fluid intake is inadequate for complete rehydration (0.5 L kg BM lost^−1^). The influence of fluid intake for those individuals performing intermittent, resistance and sport-specific exercise or undertaking cognitively demanding tasks is not as well understood, and this review serves to highlight areas for future research.

## Additional File

Additional file 1: Supplementary Table S1 - S9. (DOC 725 kb)
