# Supplementary material for: The Effect of Fluid Intake Following Dehydration on Subsequent Athletic and Cognitive Performance: a Systematic Review and Meta-analysis
Source: Sports Med Open. 2017 Mar 18;3:13. doi: 10.1186/s40798-017-0079-y (PMC5357466; doi:10.1186/s40798-017-0079-y)
Supplement: Additional file 1: — Supplementary Table S1 - S9. (DOC 725 kb) [file 40798_2017_79_MOESM1_ESM.doc]

**Additional file**

**Table S1.** Methodological quality assessment summary and Rosendal Score of included studies.

| **Citation** | A clear description of the inclusion and exclusion criteria was provided | The trials were randomized | The method used to generate the random allocation sequence was described | Sample size was justified | Attempts were made to control and/or monitor pre-trial conditions | Design incorporated measures of important baseline variables | Details were provided regarding the inability of a subject to complete study requirements | Statistical methods used to compare groups for primary outcome measure(s), and methods for additional analyses were described | Both point measures and measures of variability for the primary outcome were provided | The results of between groups statistical comparisons were reported for the primary outcome measures | The method used to assess adverse effects was described | Reproducibility of the primary outcome measure(s) was reported | A familiarization was conducted (includes VO2 max tests, if comparable to the performance task) | **Total Score (%)** |
| --- | --- | --- | --- | --- | --- | --- | --- | --- | --- | --- | --- | --- | --- | --- |
| Below et al. (1994) [62] | 0 | 0 | - | 0 | 1 | 1 | - | 1 | 1 | 1 | 0 | 0 | 1 | 55 |
| Melin et al. (1994) [47] | 0 | 0 | - | 0 | 0 | 1 | - | 1 | 1 | 1 | 0 | 0 | 1 | 45 |
| Walsh et al. (1994) [33] | 0 | 1 | 0 | 0 | 1 | 1 | - | 1 | 1 | 1 | 0 | 0 | 1 | 58 |
| McConell et al. (1997) [24] | 0 | 1 | 0 | 0 | 1 | 1 | - | 1 | 1 | 1 | 0 | 0 | 1 | 58 |
| Castellani et al. (1997) [62] | 0 | 1 | 0 | 0 | 1 | 1 | 1 | 1 | 1 | 1 | 1 | 0 | 1 | 69 |
| Greiwe et al. (1998) [52] | 1 | 0 | - | 0 | 0 | 1 | 0 | 1 | 1 | 1 | 0 | 0 | 1 | 50 |
| Montain et al. (1998) [66] | 0 | 1 | 0 | 0 | 0 | 1 | - | 1 | 1 | 1 | 0 | 0 | 1 | 50 |
| Maxwell et al. (1999) [65] | 0 | 0 | - | 0 | 1 | 1 | - | 1 | 1 | 1 | 0 | 0 | 1 | 55 |
| McConell et al. (1999) [25] | 0 | 1 | 0 | 0 | 1 | 1 | - | 1 | 1 | 1 | 0 | 0 | 1 | 58 |
| Casa et al. (2000) [76] | 1 | 1 | 0 | 0 | 1 | 1 | 1 | 1 | 1 | 1 | 1 | 0 | 1 | 77 |
| Bigard et al. (2001) [53] | 0 | 1 | 0 | 0 | 0 | 1 | - | 1 | 1 | 1 | 0 | 0 | 1 | 50 |
| Cian et al. (2001) [55] | 0 | 1 | 0 | 0 | 1 | 1 | - | 1 | 1 | 1 | 0 | 1 | 0 | 58 |
| Devlin et al. (2001) [71] | 0 | 1 | 0 | 0 | 1 | 1 | - | 1 | 1 | 1 | 0 | 0 | 1 | 58 |
| Schoffstall et al. (2001) [54] | 0 | 0 | - | 0 | 1 | 1 | - | 1 | 1 | 1 | 1 | 0 | 1 | 64 |
| Grego et al. (2005) [48] | 0 | 0 | - | 0 | 1 | 1 | - | 1 | 1 | 1 | 0 | 0 | 0 | 45 |

| Cheuvront, et al. (2005) [42] | 0 | 0 | - | 0 | 1 | 1 | - | 1 | 1 | 1 | 0 | 0 | 1 | 55 |
| --- | --- | --- | --- | --- | --- | --- | --- | --- | --- | --- | --- | --- | --- | --- |
| Cheuvront, et al. (2006) [51] | 0 | 0 | - | 0 | 1 | 1 | - | 1 | 1 | 1 | 0 | 1 | 1 | 64 |
| Hasegawa et al. (2006) [64] | 0 | 0 | - | 0 | 1 | 1 | - | 1 | 1 | 1 | 0 | 0 | 1 | 55 |
| Kavouras et al. (2006) [77] | 1 | 1 | 0 | 0 | 1 | 1 | - | 1 | 1 | 1 | 1 | 0 | 1 | 75 |
| Kenefick et al. (2006) [36] | 0 | 1 | 0 | 0 | 1 | 1 | 1 | 1 | 1 | 1 | 1 | 0 | 1 | 69 |
| Serwah et al. (2006) [68] | 1 | 1 | 0 | 0 | 1 | 1 | 1 | 1 | 1 | 1 | 0 | 0 | 0 | 62 |
| Edwards et al. (2007) [58] | 0 | 1 | 0 | 0 | 1 | 1 | - | 1 | 1 | 1 | 0 | 0 | 0 | 50 |
| Adam et al. (2008) [56] | 1 | 0 | - | 0 | 1 | 1 | - | 1 | 1 | 1 | 0 | 0 | 1 | 64 |
| Del Coso et al. (2008) [46] | 0 | 1 | 0 | 0 | 1 | 1 | - | 1 | 1 | 1 | 0 | 0 | 1 | 58 |
| D’Anci et al. (2009) [73] | 0 | 1 | 0 | 0 | 1 | 1 |  | 1 | 1 | 1 | - | 0 | 0 | 55 |
| Maxwell et al. (2009) [22] | 0 | 1 | 0 | 0 | 1 | 1 | - | 1 | 1 | 1 | 0 | 0 | 1 | 58 |
| Paik et al. (2009) [50] | 1 | 1 | 0 | 0 | 1 | 1 | - | 1 | 1 | 1 | 0 | 0 | 1 | 67 |
| Erkmen et al. (2010) [61] | 1 | 0 | - | 0 | 1 | 1 | - | 1 | 1 | 1 | 0 | 1 | 1 | 73 |
| Kenefick et al. (2010) [41] | 0 | 1 | 0 | 0 | 1 | 1 |  | 1 | 1 | 1 | 1 | 0 | 1 | 67 |
| Kraft et al. (2010) [74] | 0 | 0 | - | 0 | 1 | 1 | - | 1 | 1 | 1 | 1 | 0 | 0 | 55 |
| Ali et al. (2011) [35] | 1 | 1 | 0 | 0 | 1 | 1 |  | 1 | 1 | 1 | 0 | 0 | 0 | 58 |
| Ganio et al. (2011) [69] | 1 | 1 | 0 | 1 | 1 | 1 |  | 1 | 1 | 1 | 0 | 0 | 0 | 67 |
| Hillman et al. (2011) [29] | 1 | 1 | 0 | 0 | 1 | 1 | - | 1 | 1 | 1 | 0 | 1 | 1 | 75 |
| Kraft et al. (2011) [75] | 0 | 0 | - | 0 | 1 | 1 | - | 1 | 1 | 1 | 0 | 0 | 1 | 55 |
| Ely et al. (2012) [67] | 0 | 1 | 0 | 0 | 1 | 1 | - | 1 | 1 | 1 | 1 | 1 | 1 | 75 |
| Ali et al. (2013) [57] | 0 | 0 | - | 0 | 1 | 1 | - | 1 | 1 | 1 | 0 | 0 | 1 | 55 |
| Fritz et al. (2013) [72] | 1 | 0 | - | 0 | 1 | 1 | - | 1 | 1 | 1 | 0 | 0 | 0 | 55 |
| Owen et al. (2013) [59] | 0 | 1 | 0 | 0 | 1 | 1 | - | 1 | 1 | 1 | 0 | 1 | 1 | 67 |
| Stewart et al. (2014) [37] | 1 | 1 | 0 | 0 | 1 | 1 | - | 1 | 1 | 1 | 0 | 0 | 1 | 67 |
| Rodrigues et al. (2014) [49] | 1 | 0 | - | 0 | 1 | 1 | 1 | 1 | 1 | 1 | 1 | 1 | 1 | 83 |
| Wilson et al. (2014) [60] | 0 | 1 | 0 | 0 | 1 | 1 | - | 1 | 1 | 1 | 0 | 0 | 1 | 58 |
| Wittbrodt et al. (2015) [70] | 0 | 0 | - | 0 | 0 | 1 | - | 1 | 1 | 1 | 0 | 1 | 1 | 55 |

**Table S2.** Characteristics of research studies evaluating athletic performance on continuous exercise tasks

| **Citation, location** | **Subjects (*n*)** | **Age (y)** | **Pre- Dh BM (kg)** | **VO2 max (mL/kg/min)** | **Dh protocol; ambient temperature; RH; duration** | **Dh trial BM loss (%)** | **REC 1**  **duration** | | **Rh fluid; % fluid losses replaced; drink time** | **Mean drink rate (mL/h)** | **REC 2 duration** | **Task; ambient temperature; RH; rate of airflow** | **Performance** | | |  |
| --- | --- | --- | --- | --- | --- | --- | --- | --- | --- | --- | --- | --- | --- | --- | --- | --- |
|  |  |  |  |  |  |  |  |  |  |  |  |  | **Intervention** | **Control** | **Hedges’ *g*** | |
| **Performance duration ≥ 60 min** | | | | | | | | | | | | | | | | |
| Melin et al. (1994) [47], France | 6 M; unacclimated,  endurance trained | 26 ± 5 | 74.0 ± 4.2 | 57.5 ± 4.2 | HT; 55°C; 46%; ~2-3 h | 2.6 | | 60 min | Water (26°C); 50% (913 ± 56mL); Est. <15 min | NC | 0 | TTE treadmill marching (~50% VO2 max); 35°C; 20-30%; 0.8m/s | 112 ± 17.1 min* | 82 ± 7.3 min | 2.11 | |
| Castellani et al. (1997) [62], U.S. | 8 M; unacclimated | 22 ± 2 | 73.6 ± 6.8 | 57.9 ± 4.5 | EX (51% VO2 max); 33°C; 48%; 180 min | 4.1 | | 15 min | 0.45% NaCl + NNS (4°C); 50% (1856 ± 182mL); 45 min  (Dh trial: 100mL deionised water) | 2475 | 75 min  (1g CHO/kg BM  given at 20 min) | TTE treadmill walking (53-54% VO2 max) (max. 90 min); 36°C; 47%; 2.3m/s | 84.2 ± 6.5 min* | 58.9 ± 23.8 min | 1.37 | |
| Kenefick et al. (2006) [36], U.S. | 8M; unacclimated | 21 ± 2 | 75.2 ± 10.5 | 63.7 ± 10.2 | EX (47% VO2 max); 36°C; 43%; 75 min | 2.3 | | 10 min | 0.45% NaCl + NNS (15°C); 100% (1790mL); 20 min | 5370 | 0 | TTE treadmill running (49% VO2 max) (max. 75 min); 37°C; 42% | 70.6 ± 23.3 min* | 38.7 ± 76.5 min | 0.53 | |
| **Performance duration ~30 – 60 min** | | | | | | | | | | | | | | | | |
| Casa et al. (2000) [76], U.S. | 8 M; unacclimated,  endurance trained cyclists | 24 ± 3 | 70.1 ± 2.8 | 61.4 ± 2.3 | FR 24 h + self-administered EX (~2 h moderate intensity) 1 d prior to performance | 3.9 | | N/A | 0.45% NaCl + NNS (10°C); 50% (1405 ± 317mL); 20 min | 4215 | Est. <15 min | TTE ergometer cycling (~74% VO2 max); 37°C; 2.3m/s | 34.9 ± 11.3 min* | 19.0 ± 7.6 min | 1.56 | |
| Cheuvront et al. (2005a) [42], U.S. | 8 (6M); physically active | 24 ± 6 | 72.9 ± 11.1 | 48 ± 9 | HT; 45°C; 50%; 3 h | 2.9 | | Concurrent  Rh & Dh | Fluid NS; % replaced NS (BM deficit was 0.4% post-Rh); 3 h | NC | 3.5 h  (200mL water + recovery snack) | TW ergometer cycling 30 min; 20°C; 50%; 1m/s | 272.7 ± 53.9 kJ* | 251.8 ± 53.3 kJ | 0.36 | |
| Cheuvront et al. (2005b) [42], U.S. | 8 (6M); physically active | 24 ± 6 | 72.9 ± 11.1 | 48 ± 9 | HT; 45°C; 50%; 3 h | 3.0 | | Concurrent  Rh & Dh | Fluid NS; % replaced NS (BM deficit was 0.4% post-Rh); 3 h | NC | 3.5 h  (200mL water + recovery snack) | TW ergometer cycling 30 min; 2°C; 50%; 2.2m/s | 277.3 ± 64.5 kJ | 269.6 ± 63.6 kJ | 0.11 | |
| Paik et al. (2009) [50], South Korea | 10 M; moderately active | 26 ± 3 | 69.0 ± 7.7 | 53.6 ± 11.4 | HT; ~3 h | ~3.0 | | NS | Water; Est.100%; 2 h | NC | NS | TTE treadmill running (~80% VO2 max) | 32.2 ± 8.9 min | 28.0 ± 6.0 min | 0.53 | |
| **Performance duration ~15 – 30 min** | | | | | | | | | | | | | | | | |
| McConell et al. (1999a) [25], Australia | 8 M; well-trained cyclists/triathletes | 26 ± 3 | 78.8 ± 9.3 | 63.8 ± 1.2 | EX (80% VO2 max); 21°C; 41%; 45 min | 1.9 | | Concurrent  Rh & Dh | Water; 100% (1470 ± 141mL); 45 min | 1960 | 0 | TW ergometer cycling 15 min; 21°C; 41%; airflow rate NS | 269 ± 25.4 kJ | 273 ± 22.6 kJ | 0.16 | |
| McConell et al. (1999b) [25], Australia | 8 M; well-trained cyclists/triathletes | 26 ± 3 | 79.3 ± 9.9 | 63.8 ± 1.2 | EX (80% VO2 max); 21°C; 41%; 45 min | 1.9 | | Concurrent  Rh & Dh | Water; 50% (720 ± 85mL); 45 min | 960 | 0 | TW ergometer cycling 15 min; 21°C; 41%; airflow rate NS | 267 ± 22.6 kJ | 273 ± 22.6 kJ | 0.25 | |
| Kavouras et al. (2006) [77], U.S. | 8 M; acclimated endurance trained cyclists | 24 ± 3 | 71.8 ± 3.1 | 61.4±2.3 | FR 24 h + self-administered EX (~2 h moderate intensity) 1 d prior to performance | 3.9 | | N/A | Water + NNS (10°C); ~75% (2142 ± 113mL); 80 min | 1607 | 30 min | TTE ergometer cycling (~74% VO2 max); 37°C; 48%; 2.54m/s | 27.1 ± 9.3 min* | 19.0 ± 7.6 min | 0.90 | |
| Kenefick et al. (2010a) [41], U.S. | 8 M | 23 ± 5 | 87.3 ± 13.7 | 43.6 ± 4.1 | EX (walking 4.8km∙h-1); 50°C; 20%; 3 h work/rest cycle | 4.1 | | Concurrent  Rh & Dh | 0.05% NaCl; ~105% (3700 ± 600mL); 3 h | 1233 | 2 h | TW ergometer cycling 15 min; 10°C | 199 ± 37 kJ | 194 ± 36 kJ | 0.13 | |
| Kenefick et al. (2010b) [41], U.S. | 8 M | 23 ± 3 | 79.8 ± 4.9 | 45.3 ± 4.6 | EX (walking 4.8km∙h-1); 50°C; 20%; 3 h work/rest cycle | 4.2 | | Concurrent  Rh & Dh | 0.05% NaCl; ~105% (3700 ± 600mL); 3 h | 1233 | 2 h | TW ergometer cycling 15 min; 20°C | 198 ± 22 kJ | 179 ± 20 kJ | 0.85 | |

| Kenefick et al. (2010c) [41], U.S. | 8 M | 24 ± 6 | 85.4 ± 14.5 | 46.3 ± 5.2 | EX (walking 4.8km∙h-1); 50°C; 20%; 3 h work/rest cycle | 4.0 | Concurrent  Rh & Dh | 0.05% NaCl; ~105% (3700 ± 600mL); 3 h | 1233 | 2 h | TW ergometer cycling 15 min; 30°C | 198 ± 21kJ* | 174 ± 30 kJ | 0.88 |  | | | | | | | | | | |
| --- | --- | --- | --- | --- | --- | --- | --- | --- | --- | --- | --- | --- | --- | --- | --- | --- | --- | --- | --- | --- | --- | --- | --- | --- | --- |
| Kenefick et al. (2010d) [41], U.S. | 8 M | 22 ± 3 | 89.1 ± 6.7 | 43.7 ± 7.0 | EX (walking 4.8km∙h-1); 50°C; 20%; 3 h work/rest cycle | 4.1 | Concurrent  Rh & Dh | 0.05% NaCl; ~105% (3700 ± 600mL); 3 h | 1233 | 2 h | TW ergometer cycling 15 min; 40°C | 157 ± 19 kJ* | 122 ± 37 kJ | 1.13 |  | | | | | | | | | | |
| **Performance duration ~5 – 15 min** | | | | | | | | | | | | | | | |  |  |  |  |  |  |  |  |  |  |
| Below et al. (1994) [63], U.S. | 8 M; acclimated endurance trained | 23 ± 3 | 70.6 ± 8.5 | 62.9 ± 2.8 | EX (80% VO2 max); 31°C; 54%; 50 min | 2.0 | Concurrent  Rh & Dh | 0.46% Na+ + NNS (37-39°C); ~100% (1330 ± 170mL); 50 min  (Dh trial: 200mL water ingested) | 1330 | 0 | TT ergometer cycling to target amount of work; 31°C; 54% | 10.5 ± 0.8 min* | 11.3 ± 0.8 min | 0.95 |  | | | | | | | | | | |
| Walsh et al. (1994a) [33], South Africa | 6 M; endurance  trained cyclists/ triathletes | 26 ± 4 | 71.7 ± 7.7 | 61.4 ± 4.4 | EX (70% VO2 max) ; 30°C; 60%; 60 min | 1.8 | Concurrent  Rh & Dh | NaCl + NNS; % replaced NS (1000mL); 50 min  (Dh trial: 10mL water mouth rinse) | 1200 | < 90 s | TTE ergometer cycling (~90% VO2 max); 30°C; 60%; 0.8m/s | 9.8 ± 3.9 min* | 6.5 ± 3.0 min | 0.87 |  | | | | | | | | | | |
| Hillman et al. (2011a) [29], U.K. | 7 M; unacclimated competitive cyclists | 36 ± 6 | 72.8 ± 7.0 | NS | EX (95% lactate threshold); 23°C; 90 min | 3.0 | Concurrent  Rh & Dh | Water; 100%; 90 min | NC | 15 min | Power output ergometer cycling 5km TT; 23°C | 282 ± 37.0 W | 268 ± 31.7 W | 0.38 |  | | | | | | | | | | |
| Hillman et al. (2011b) [29], U.K. | 7 M; unacclimated competitive cyclists | 36 ± 6 | 72.8 ± 7.0 | NS | EX (95% lactate threshold); 34°C; 90 min | 3.8 | Concurrent  Rh & Dh | Water; 100%; 90 min | NC | 15 min | Power output ergometer cycling 5 km TT; 34°C | 262 ± 42.3 W* | 229 ± 31.7 W | 0.83 |  | | | | | | | | | | |
| Stewart et al. (2014) [37], Australia | 7 M; recreationally active | 23 ± 4 | 80.6 ± 10.2 | 52.7 ± 7.9 | EX (50-65% PPO); 37°C; 65%; 120 min | 3.8 | 0 | Water; 115% (3000mL); 90 min | 1500 | 30 min | TT ergometer cycling 5km; 18-25°C; 20-30% | 7.1 ± 1.3 min | 7.3 ± 1.5 min | 0.13 |  | | | | | | | | | | |
| **Performance duration ~1 – 5 min** | | | | | | | | | | | | | | | |  | | | | | | | | | |
| McConell et al. (1997a) [24], Australia | 7 M; well-trained cyclists/triathletes | 24 ± 3 | 68.6 ± 7.4 | 68.4 ± 2.5 | EX (69% VO2 max); 21°C, 43%; 120 min | 3.2 | Concurrent  Rh & Dh | Deionised water; 50% (1160 ± 130mL); 120 min | 580 | 0 | TTE ergometer cycling (90% VO2 max); 21°C, 43%; airflow rate NS | 248 ± 283 s | 171 ± 198 s | 0.30 |  | | | | | | | | | | |
| McConell et al. (1997b) [24], Australia | 7 M; well-trained cyclists/triathletes | 24 ± 3 | 68.3 ± 7.9 | 68.4± 2.5 | EX (69% VO2 max); 21°C, 43%; 120 min | 3.2 | Concurrent  Rh & Dh | Deionised water; 100% (2320 ± 260mL); 120 min | 1160 | 0 | TTE ergometer cycling (90% VO2 max); 21°C, 43%; airflow rate NS | 328 ± 246 s* | 171 ± 198 s | 0.66 |  | | | | | | | | | | |
| Hasegawa et al. (2006) [64], Japan | 9 M; untrained | 22 ± 2 | 61.7 ± 6.3 | 48.5 ± 4.5 | EX (~60% VO2 max); 32°C; 80%; 60 min | Est. ~1.6 | Concurrent  Rh & Dh | Water (14-16°C); 100%; 60 min | NC | 4 min | TTE ergometer cycling (~80% VO2 max); 32°C; 80% | 373 ± 51.0 s* | 152 ± 48.0 s | 4.25 |  | | | | | | | | | | |

**Table S3.** Characteristics of research studies evaluating athletic performance on intermittent exercise tasks

| **Citation, location** | **Subjects (*n*)** | **Age (y)** | **Pre- Dh BM (kg)** | **VO2 max (mL/kg/min)** | | | **Dh protocol; ambient temperature; RH; duration** | | **Dh trial BM loss (%)** | **REC 1**  **duration** | | | **Rh fluid; % fluid losses replaced; drink time** | | **Mean drink rate (mL/h)** | **REC 2 duration** | | **Task; ambient temperature; RH; rate of airflow** | | **Performance** | | | |  |
| --- | --- | --- | --- | --- | --- | --- | --- | --- | --- | --- | --- | --- | --- | --- | --- | --- | --- | --- | --- | --- | --- | --- | --- | --- |
|  |  |  |  |  |  |  |  |  |  |  |  |  |  |  |  |  |  |  |  | **Intervention** | | **Control** | **Hedges’ *g*** | |
| Devlin et al. (2001a) [71], Australia | 7 M; sub-elite cricketers | 21 ± 1 | 89 ± 13 | | 56 ± 6 | EX; 28°C; 40%; 60 min W/R | | 2.8 | | | Concurrent  Rh & Dh | Water; 80% (2240±270mL); 60 min | | 2240 | | | 0 | | 20 m shuttle runs MMRT[[1]](#footnote-2); 16°C, 60% | | 82.6 ± 20.8*n** | 76.2 ± 19.3*n* | 0.30 | |
|  |  |  |  |  |  |  |  |  |  |  |  | (Dh trial: 180mL frozen water) | |  |  |  |  |  |  |  |  |  |  |  |
| Cheuvront, et al. (2006) [51], U.S. | 8 M; physically  active | 28 ± 5 | 77.5 ± 10.8 | | 52 ± 6 | HT; 45°C; 50%; 180 min | | 2.7 | | | Concurrent  Rh & Dh | Water; Est. 100%; 180 min | | - | | | Testing 5,  30 & 60  min post-Dh | | 15 s WAnT ergometer cycling;  22°C, 65% | |  |  |  | |
|  |  |  |  |  |  |  |  |  |  |  |  |  |  |  |  |  |  |  | Abs. mean power output | | 775 ± 121 W | 770 ± 127 W | 0.04 | |
|  |  |  |  |  |  |  |  |  |  |  |  |  |  |  |  |  |  |  | Rel. mean power output | | 9.9 ± 1.0 W | 10.1 ± 1.3 W | 0.16 | |
|  |  |  |  |  |  |  |  |  |  |  |  |  |  |  |  |  |  |  | Abs. PPO | | 888 ± 123 W | 884 ± 153 W | 0.03 | |
|  |  |  |  |  |  |  |  |  |  |  |  |  |  |  |  |  |  |  | Rel. PPO | | 11.4 ± 1.0 W | 11.7 ± 1.3 W | 0.24 | |
|  |  |  |  |  |  |  |  |  |  |  |  |  |  |  |  |  |  |  | Rate of fatigue | | No effect (mean±SD unpublished) | | | |
| Walsh et al. (1994b) [33], South Africa | 6 M; endurance  trained cyclists/ triathletes | 26 ± 4 | 71.7 ± 7.7 | | 61.4 ± 4.4 | EX (70% VO2 max); 30°C; 60%; 60 min | | 1.8 | | | Concurrent  Rh & Dh | NaCl + NNS; % replaced NS (1000mL); 50 min  (Dh trial: 10mL water mouth rinse) | | 1200 | | | ~15 min | | IST ergometer cycling[[2]](#footnote-3) | |  |  |  | |
|  |  |  |  |  |  |  |  |  |  |  |  |  |  |  |  |  |  |  | Max. speed | | 14.4 ± 2.2 m/s | 13.9 ± 1.4 m/s | 0.25 | |
|  |  |  |  |  |  |  |  |  |  |  |  |  |  |  |  |  |  |  | Power output | | 165.9 ± 10.7 N | 166.0 ± 32.6 N | <0.01 | |
| Maxwell et al. (1999) [65], U.K. | 11 M; untrained | 26 ± 5 | 77.5 ± 23.0 | | NS | EX (72% predicted max. HR); 32°C; 73%; 48 min | | 1.5 | | | Concurrent  Rh & Dh | 0.50% NaCl + NNS (5°C); 155% (1834±195); 88 min | | 1250 | | | 120 min | | Total treadmill sprint time MART[[3]](#footnote-4); 32°C, 73% | | 154 ± 30 s* | 148 ± 30 s | 0.19 | |
| Edwards et al. (2007a) [58], N.Z. | 11 M; moderately active soccer  players | 24 ± 3 | 74.0 ± 10.5 | | 50.9 ± 4.0 | EX (soccer match + 90% ventilator threshold); 19-25°C; 46-57%; 90 min | | 2.4 | | | Concurrent  Rh & Dh | Water (20-24°C); 80%; 90min | | NC | | | 0 | | Total distance run Yo-Yo Intermittent Recovery Test[[4]](#footnote-5) | | Rh sig. increased total distance run (mean±SD unpublished) | | | |
| Maxwell et al. (2009a) [22], U.K. | 8 M; unacclimated game players | 23 ± 6 | 76.8 ± 7.7 | | 59.9 ± 8.0 | EX (IST + ~50% VO2 max); 36°C; 49%; 90 min | | 3.9 | | | 0 | Water; 150% + 0.5 L PM & 0.5 L AM; timeframe NS | | NC | | | Overnight | | IST ergometer cycling[[5]](#footnote-6); 36°C; 49% | |  | | | |
|  |  |  |  |  |  |  |  |  |  |  |  |  |  |  |  |  |  |  | Total work | | 3790 ± 556 kJ | 3647 ± 339 kJ | 0.29 | |
|  |  |  |  |  |  |  |  |  |  |  |  |  |  |  |  |  |  |  | Abs. PPO | | 1315 ± 129 W | 1282 ± 128 W | 0.24 | |
|  |  |  |  |  |  |  |  |  |  |  |  |  |  |  |  |  |  |  | Rel. PPO | | 17.3 ± 1.7 W | 17.3 ± 1.9 W | <0.01 | |
|  |  |  |  |  |  |  |  |  |  |  |  |  |  |  |  |  |  |  | RSB 1: | |  |  |  | |
|  |  |  |  |  |  |  |  |  |  |  |  |  |  |  |  |  |  |  | Total work | | 3396 ± 558 kJ | 3431 ± 389 kJ | 0.07 | |
|  |  |  |  |  |  |  |  |  |  |  |  |  |  |  |  |  |  |  | Abs. PPO | | 1178 ± 257 W | 1194 ± 121 W | 0.08 | |
|  |  |  |  |  |  |  |  |  |  |  |  |  |  |  |  |  |  |  | Rel. PPO | | 16.4 ± 1.5 W | 16.1 ± 1.4 W | 0.20 | |
|  |  |  |  |  |  |  |  |  |  |  |  |  |  |  |  |  |  |  | RSB 2: | |  |  |  | |
|  |  |  |  |  |  |  |  |  |  |  |  |  |  |  |  |  |  |  | Total work | | 3463 ± 273 kJ* | 3189 ± 261 kJ | 0.97 | |
|  |  |  |  |  |  |  |  |  |  |  |  |  |  |  |  |  |  |  | Abs. PPO | | 1227 ± 108 W­* | 1125 ± 134 W | 0.79 | |
|  |  |  |  |  |  |  |  |  |  |  |  |  |  |  |  |  |  |  | Rel. PPO | | 16.2 ± 0.7 W | 15.1 ± 1.4 W | 0.94 | |

| Maxwell et al. (2009b) [22], U.K. | 8 M; unacclimated game players | 23 ± 6 | 76.8 ± 7.7 | 59.9 ± 8.0 | EX (IST + ~50% VO2 max); 36°C; 49%; 90 min | 3.9 | 0 | Water; 100%; timeframe NS | NC | Overnight | IST ergometer cycling[[6]](#footnote-7); 36°C; 49% |  |  |  |
| --- | --- | --- | --- | --- | --- | --- | --- | --- | --- | --- | --- | --- | --- | --- |
|  |  |  |  |  |  |  |  |  |  |  | Total work | 3785 ± 628 kJ | 3647 ± 339 kJ | 0.26 |
|  |  |  |  |  |  |  |  |  |  |  | Abs. PPO | 1304 ± 175 W | 1282 ± 128 W | 0.13 |
|  |  |  |  |  |  |  |  |  |  |  | Rel. PPO | 17.2 ± 1.8 W | 17.3 ± 1.9 W | 0.05 |
|  |  |  |  |  |  |  |  |  |  |  | RSB 1: |  |  |  |
|  |  |  |  |  |  |  |  |  |  |  | Total work | 3551 ± 556 kJ | 3431 ± 389 kJ | 0.24 |
|  |  |  |  |  |  |  |  |  |  |  | Abs. PPO | 1211 ± 167 W | 1194 ± 121 W | 0.11 |
|  |  |  |  |  |  |  |  |  |  |  | Rel. PPO | 16.1 ± 1.7 W | 16.1 ± 1.4 W | <0.01 |
|  |  |  |  |  |  |  |  |  |  |  | RSB 2: |  |  |  |
|  |  |  |  |  |  |  |  |  |  |  | Total work | 3205 ± 622 kJ | 3189 ± 261 kJ | 0.03 |
|  |  |  |  |  |  |  |  |  |  |  | Abs. PPO | 1167 ± 209 W­ | 1125 ± 134 W | 0.23 |
|  |  |  |  |  |  |  |  |  |  |  | Rel. PPO | 15.2 ± 1.9 W | 15.1 ± 1.4 W | 0.06 |
| Kraft et al. (2011) [75], U.S. | 10 M | 22 ± 3 | 81.9 ± 6.8 | NS | WI; 39°C; 113-133 min | 3.0 | Concurrent  Rh & Dh | Water; ~100%; 113-133 min | NC | Min. 45 min | IST ergometer cycling[[7]](#footnote-8) |  |  |  |
|  |  |  |  |  |  |  |  |  |  |  | Mean power output | 582 ± 76 W | 569 ± 72 W | 0.17 |
|  |  |  |  |  |  |  |  |  |  |  | Abs. PPO | 870 ± 128 W | 857 ± 145 W | 0.09 |
|  |  |  |  |  |  |  |  |  |  |  | Seconds >90 rev/min | 7.9 ± 1.9 s | 7.5 ± 1.6 s | 0.22 |
|  |  |  |  |  |  |  |  |  |  |  | Rate of fatigue | No effect (mean±SD unpublished) | | |
| Owen et al. (2013a) [59], U.K | 13 M; semi-professional soccer players | 22 ± 3 | 73.5 ± 4.8 | 54 ± 3 | EX (Loughborough Intermittent Shuttle Test); 19°C; 59%; 105 min | 2.5 | Concurrent  Rh & Dh | Water; 89% (1650 ± 170mL); consumed during five 3 min recovery intervals | 943 | 5 min | Total distance run Yo-Yo Intermittent Recovery Test[[8]](#footnote-9); 19°C; 59% | 345 ± 42 m | 330 ± 47 m­ | 0.33 |
| Owen et al. (2013b) [59], U.K | 13 M; semi-professional soccer players | 22 ± 3 | 73.5 ± 4.8 | 54 ± 3 | EX (Loughborough Intermittent Shuttle Test); 19°C; 59%; 105 min work/rest cycle | 2.5 | Concurrent  Rh & Dh | Water; *ad libitum* ≈ 51% (850 ± 190mL); consumed during five 3 min recovery intervals | 486 | 5 min | Total distance run Yo-Yo Intermittent Recovery Testt; 19°C; 59% | 342 ± 35 m | 330 ± 47 m­ | 0.28 |

**Table S4.** Characteristics of research studies evaluating athletic performance on resistance exercise tasks

| **Citation, location** | **Subjects (*n*)** | **Age (y)** | **Pre- Dh BM (kg)** | **VO2 max (mL/kg/min)** | **Dh protocol; ambient temperature; RH; duration** | **Dh trial BM loss (%)** | **REC 1**  **duration** | | **Rh fluid; % fluid losses replaced; drink time** | **Mean drink rate (mL/h)** | **REC 2 duration** | | **Task; ambient temperature; RH; rate of airflow** | **Performance** | | |  |
| --- | --- | --- | --- | --- | --- | --- | --- | --- | --- | --- | --- | --- | --- | --- | --- | --- | --- |
|  |  |  |  |  |  |  |  |  |  |  |  |  |  | **Intervention** | **Control** | **Hedges’ *g*** | |
| Montainet al. (1998) [66], U.S | 10 (5 M); physically active | 21-40 | 66.1 ± 13.0 | NS | EX (moderate intensity); 40°C; 20%; 2-3 h | 4.0 | | Concurrent  Rh & Dh | Water; *ad libitum*, % replaced NS (BM deficit was 0.6% post-Rh); 2-3 h | NC | | 3-8 h  (200mL fruit juice  + standardised meal) | Knee extensors:  ET >50% MVC | 251 ± 15 sec* | 213 ± 12 s | 2.70 | |
|  |  |  |  |  |  |  |  |  |  |  |  |  | MVC, pre-ET test | No effect (mean±SD unpublished) | | | |
|  |  |  |  |  |  |  |  |  |  |  |  |  | MVC, 30 sec post-ET test | Dh. increased MVC (mean±SD unpublished) | | | |
|  |  |  |  |  |  |  |  |  |  |  |  |  | MVC, >30 sec post-ET test | No effect (mean±SD unpublished) | | | |
| Greiwe et al. (1998) [52], U.S. | 7 M; unacclimated | 23 ± 1 | 82.1 ± 9.7 | NS | HT; 74-79°C; 186 min | 3.8 | | Concurrent  Rh & Dh | Water; 100%; 186 min | NC | | 120 min | Peak torque, knee extensors | 297 ± 79 N/m | 311 ± 113 N/m | 0.13 | |
|  |  |  |  |  |  |  |  |  |  |  |  |  | Peak torque, elbow flexors | 80 ± 12 N/m | 83 ± 15 N/m | 0.21 | |
|  |  |  |  |  |  |  |  |  |  |  |  |  | ET 100% MVC, knee extensors | 41.8 ± 6.0 s | 45.3 ± 7.6 s | 0.48 | |
|  |  |  |  |  |  |  |  |  |  |  |  |  | ET 100% MVC, elbow flexors | 46.0 ± 6.4 s | 44.0 ± 9.4 s | 0.23 | |
| Bigard et al. (2001) [53], France. | 11 M; unacclimated physically active | 22 ± 3 | 78.8 ± 7.6 | NS | HT; 80-85°C; 20%; max. 90 min. | 3.0 | | 60 min | 0.38% Na+ and 0.20% K+; 100% (2340mL); 120 min | 1170 | | 0 | Knee extensors:  MVC | 428 ± 73.0 N/m | 422 ± 66.3 N/m | 0.08 | |
|  |  |  |  |  |  |  |  |  |  |  |  |  | ET 70% MVC | 47 ± 13.3 s | 40 ± 13.3 s | 0.51 | |
|  |  |  |  |  |  |  |  |  |  |  |  |  | ET 25% MVC | 163 ± 56.4 s* | 150 ± 59.7 s | 0.22 | |
| Schoffstall et al. (2001) [54], U.S. | 10 M; competitive power lifters | 25 ± 3 | 85.5 ± 16.4 | NS | HT; 60°C; ~120 min. | 1.7 | | NS | Water; *ad libitum* ≈ 110% (1360 ± 480mL); 120 min | 680 | | 0 | Bench press 1 x RM | 117 ± 24.7 kg* | 111 ± 22.8 kg | 0.24 | |
| Del Coso et al. (2008) [46], Spain | 7 (gender NS); endurance trained acclimated cyclists | NS | 71.5 ± 7 | 61 ± 8 | EX (63% VO2 max); 36°C; 29%; 120 min. | 3.7 | | Concurrent  Rh & Dh | Mineral water; 100% (2400 ± 100mL); 120 min | 1200 | | 0 | MVC, knee extensor | Dh. decreased MVC (mean±SD unpublished) | | | |
| Kraft et al. (2010) [74], U.S. | 10 M; recreationally strength trained | 23 ± 3 | 81.0 ± 10.4 | NS | WI; 39°C; ~120 min | 3.1 | | Concurrent  Rh & Dh | Water; 100%; ~120 min | NC | | Min. 45 min | Full body resistance exercise protocol, total repetitions[[9]](#footnote-10) at 12RM | 169.4 ± 29.1 *n** | 144.1 ± 26.6 *n* | 0.87 | |
| Ali et al. (2013) [57], N.Z. | 10 M; university-  level soccer players | 24 ± 2 | 78.7 ± 9.4 | NS | EX (Loughborough Intermittent Shuttle Test);  22°C; 62-64%; 90 min | 2.9 | | Concurrent  Rh & Dh | Water; ~50% (~1338mL); consumed during five 3 min recovery intervals | NC | | 0 | MVC (3.14 rad/s), knee extensor |  |  |  | |
|  |  |  |  |  |  |  |  |  |  |  |  |  | Peak torque | 138 ± 27.9 N/m | 139 ± 19.3 N/m | 0.04 | |
|  |  |  |  |  |  |  |  |  |  |  |  |  | Total work | 166 ± 42.1 J | 178 ± 29.2 J | 0.31 | |
|  |  |  |  |  |  |  |  |  |  |  |  |  | Mean power output | 238 ± 54.7 W | 257 ± 36.5 W | 0.39 | |
|  |  |  |  |  |  |  |  |  |  |  |  |  | MVC (1.05 rad/s), knee extensor |  |  |  | |
|  |  |  |  |  |  |  |  |  |  |  |  |  | Peak torque | 202 ± 37.2 N/m | 194 ± 31.9 N/m | 0.22 | |
|  |  |  |  |  |  |  |  |  |  |  |  |  | Total work | 235 ± 45.5 J | 226 ± 41.9 J | 0.19 | |
|  |  |  |  |  |  |  |  |  |  |  |  |  | Mean power output | 127 ± 16.0 W | 125 ± 20.4 W | 0.10 | |
|  |  |  |  |  |  |  |  |  |  |  |  |  | MVC, knee extensors |  |  |  | |
|  |  |  |  |  |  |  |  |  |  |  |  |  | Peak torque | 199 ± 47.7 N/m | 207 ± 62.2 N/m | 0.14 | |
|  |  |  |  |  |  |  |  |  |  |  |  |  | Mean torque | 169 ± 39.7 N/m | 177 ± 53.5 N/m | 0.16 | |
|  |  |  |  |  |  |  |  |  |  |  |  |  | MVC, knee flexors |  |  |  | |
|  |  |  |  |  |  |  |  |  |  |  |  |  | Peak torque | 73.5 ± 27.2 N/m | 65.3 ± 24.6 N/m | 0.30 | |
|  |  |  |  |  |  |  |  |  |  |  |  |  | Mean torque | 79.4 ± 27.8 N/m | 69.9 ± 26.0 N/m | 0.33 | |
| Wilson et al. (2016) [60], U.K. | 8 M; licenced jockeys | 24 ± 3 | 65.7 ± 7.4 | NS | EX (jogging 10km/h), 20°C (wearing sweat suit); 45 min | 1.8 | | Concurrent  Rh & Dh | Water; ~100% (700mL); boluses consumed half-way and immediately post-EX | - | | Est. <15 min | Max. strength, chest | -0.62 ± 1.0 % Δ* | -13.8 ± 3.0 % Δ | 5.57 | |
|  |  |  |  |  |  |  |  |  |  |  |  |  | Max. strength, legs  Data is % change from baseline performance | -0.56 ± 2.5 % Δ* | -4.8 ± 4.8 % Δ | 1.05 | |
| Rodrigues et al. (2014) [49], Brazil | 10 M; unacclimated recreationally  active | 23 ± 2 | 77.9 ± 7.4 | NS | EX (~70-75% predicted max. HR); 36-37°C; 45%; 91 min | 2.0 | | Concurrent  Rh & Dh | Water; % replaced NS (BM deficit was 0.2% post-Rh); 91 min | NC | | 30 min | Peak torque, knee extensors  Peak torque, elbow flexors | 282 ± 38.7 N/m*  67.9 ± 13.9 N/m | 247 ± 40.5 N/m  63.0 ± 13.6 N/m | 0.85  0.34 | |

**Table S5.** Characteristics of research studies evaluating athletic performance on sport-specific exercise tasks

| **Citation, location** | **Subjects (*n*)** | **Age (y)** | **Pre- Dh BM (kg)** | **VO2 max (mL/kg/min)** | **Dh protocol; ambient temperature; RH; duration** | **Dh trial BM loss (%)** | **REC 1**  **duration** | | **Rh fluid; % fluid losses replaced; drink time** | **Mean drink rate (mL/h)** | **REC 2 duration** | | **Task; ambient temperature; RH; rate of airflow** | **Performance** | |  |
| --- | --- | --- | --- | --- | --- | --- | --- | --- | --- | --- | --- | --- | --- | --- | --- | --- |
|  |  |  |  |  |  |  |  |  |  |  |  |  |  | **Intervention** | **Control** | **Hedges’ *g*** |
| Devlin et al. (2001b) [71], Australia | 7 M; sub-elite cricketers | 21 ± 1 | 89 ± 13 | 56 ± 6 | EX; 28°C; 40%; 60 min W/R | 2.8 | | Concurrent  Rh & Dh | Water; 80% (2240±270mL); 60 min  (Dh trial: 180mL frozen water) | 2240 | | 0 | 16°C; 60%  Bowling accuracy, line[[10]](#footnote-11) | 2.9 ± 0.5* | 3.4 ± 0.6 | 0.85 |
|  |  |  |  |  |  |  |  |  |  |  |  |  | Bowling accuracy, lengthv | 2.9 ± 0.5* | 3.4 ± 0.6 | 0.85 |
|  |  |  |  |  |  |  |  |  |  |  |  |  | Bowling velocity | 104.8 ± 4.1 km/hr | 101.5 ± 4.1 km/hr | 0.75 |
| Ali et al. (2011) [35], N.Z. | 10 (0 M); soccer players | 26 ± 5 | 63.5 ± 5.7 | 47 ± 4 | EX (Loughborough Intermittent Shuttle Test);  17-18°C; 58-62%; 90 min | 1.4 | | Concurrent  Rh & Dh | Water; ~107% (~953mL); consumed during five 3 min recovery intervals | NC | | 0 | Loughborough Passing Test[[11]](#footnote-12) |  |  |  |
|  |  |  |  |  |  |  |  |  |  |  |  |  | Movement time | 50.5 ± 5.9 s | 51.5 ± 5.5 s | 0.17 |
|  |  |  |  |  |  |  |  |  |  |  |  |  | Penalty time | 31.0 ± 11.1 s | 34.8 ± 9.3 s | 0.45 |
|  |  |  |  |  |  |  |  |  |  |  |  |  | Total performance time | 81.5 ± 14.3 s | 86.3 ± 14.1 s | 0.32 |
| Fritz et al. (2013) [72], South Africa | 13 M; elite squash players | 24 ± 3 | 74.7 ± 7.8 | NS | EX (x2 squash matches) | 1.3 | | Concurrent  Rh & Dh | Water (~20°C); % replaced NS (400mL); consumed during two 90 s match breaks | NC | | NS | TT on Ghosting Test[[12]](#footnote-13) | 66.5 ± 6.1 s* | 63.6 ± 3.8 s | 0.55 |
| Owen et al. (2013c) [59], U.K | 13 M; semi-professional soccer players | 22 ± 3 | 73.5 ± 4.8 | 54 ± 3 | EX (Loughborough Intermittent Shuttle Test); 19°C; 59%; 105 min | 2.5 | | Concurrent  Rh & Dh | Water; 89% (1650 ± 170mL); consumed during five 3 min recovery intervals | 943 | | 5 min | 19°C; 59%  Loughborough Passing Testw |  |  |  |
|  |  |  |  |  |  |  |  |  |  |  |  |  | Movement time | 47.5 ± 2.7 s | 47.9 ± 4.1 s | 0.11 |
|  |  |  |  |  |  |  |  |  |  |  |  |  | Penalty time | 5.8 ± 2.0 s | 6.7 ± 2.1 s | 0.43 |
|  |  |  |  |  |  |  |  |  |  |  |  |  | Total performance time | 53.3 ± 3.8 s | 54.6 ± 4.2 s | 0.31 |
|  |  |  |  |  |  |  |  |  |  |  |  |  | Loughborough Shooting Testw |  |  |  |
|  |  |  |  |  |  |  |  |  |  |  |  |  | Time taken | 8.2 ± 0.3 s | 8.2 ± 0.3 s | 0.00 |
|  |  |  |  |  |  |  |  |  |  |  |  |  | Shot speed | 75.1 ± 4.2 km/h | 76.4 ± 5.2 km/h | 0.29 |
|  |  |  |  |  |  |  |  |  |  |  |  |  | Points per shot | 1.3 ± 0.6 *n* | 1.2 ± 0.6 *n* | 0.16 |
| Owen et al. (2013d) [59], U.K | 13 M; semi-professional soccer players | 22 ± 3 | 73.5 ± 4.8 | 54 ± 3 | EX (Loughborough Intermittent Shuttle Test); 19°C; 59%; 105 min work/rest cycle | 2.5 | | Concurrent  Rh & Dh | Water; *ad libitum* ≈ 51% (850 ± 190mL); consumed during five 3 min recovery intervals | 486 | | 5 min | 19°C; 59%  Loughborough Passing Testw |  |  |  |
|  |  |  |  |  |  |  |  |  |  |  |  |  | Movement time | 48.4 ± 2.3 s | 47.9 ± 4.1 s | 0.15 |
|  |  |  |  |  |  |  |  |  |  |  |  |  | Penalty time | 6.8 ± 2.2 s | 6.7 ± 2.1 s | 0.05 |
|  |  |  |  |  |  |  |  |  |  |  |  |  | Total performance time | 55.3 ± 3.2 s | 54.6 ± 4.2 s | 0.18 |
|  |  |  |  |  |  |  |  |  |  |  |  |  | Loughborough Shooting Testw |  |  |  |
|  |  |  |  |  |  |  |  |  |  |  |  |  | Time taken | 8.2 ± 0.3 s | 8.2 ± 0.3 s | 0.00 |
|  |  |  |  |  |  |  |  |  |  |  |  |  | Shot speed | 76.1 ± 4.9 km/h | 76.4 ± 5.2 km/h | 0.06 |
|  |  |  |  |  |  |  |  |  |  |  |  |  | Points per shot | 1.1 ± 0.5 *n* | 1.2 ± 0.6 *n* | 0.18 |
| Wilson et al. (2016) [60], U.K. | 8 M; licenced jockeys | 24 ± 3 | 65.7 ± 7.4 | NS | EX (jogging 10km/h), 20°C (wearing sweat suit); 45 min | 1.8 | | Concurrent  Rh & Dh | Water; ~100% (700mL); boluses consumed half-way and immediately post-EX | - | | Est. <15 min | Pushing frequency in the final 24 s of a simulated race ride  All data is % change from baseline performance | -0.07 ± 1.5 % Δ* | -2.8 ± 2.0 % Δ | 1.46 |

**Table S6.** Characteristics of research studies evaluating athletic performance on balance tasks

| **Citation, location** | **Subjects (*n*)** | **Age (y)** | **Pre- Dh BM (kg)** | **VO2 max (mL/kg/min)** | **Dh protocol; ambient temperature; RH; duration** | **Dh trial BM loss (%)** | **REC 1**  **duration** | | **Rh fluid; % fluid losses replaced; drink time** | **Mean drink rate (mL/h)** | **REC 2 duration** | | **Task; ambient temperature; RH; rate of airflow** | **Performance** | | | **Hedges’** |
| --- | --- | --- | --- | --- | --- | --- | --- | --- | --- | --- | --- | --- | --- | --- | --- | --- | --- |
|  |  |  |  |  |  |  |  |  |  |  |  |  |  | **Intervention** | **Control** | ***g*** | |
| Erkmen et al. (2010) [61], Turkey | 17 M; physically active | 22 ± 2 | 69.5 ± 9.2 | NS | EX (75-85% HR max); 21-24°C; 60 min | 2.2 | | Concurrent  Rh & Dh | Water; 100%; 60 min | - | | Testing 0 & 20 min post-Dh | One-leg stand static balance test |  |  |  | |
|  |  |  |  |  |  |  |  |  |  |  |  |  | Eyes open OSI[[13]](#footnote-14) (0 min) | 2.71 ± 0.69* | 4.13 ± 1.63 | 1.12 | |
|  |  |  |  |  |  |  |  |  |  |  |  |  | Eyes closed OSI(0 min) | 3.39 ±1.02 | 4.14 ± 1.01 | 0.72 | |
|  |  |  |  |  |  |  |  |  |  |  |  |  | Eyes open OSI (20 min) | 2.65 ± 1.10 | 2.93 ± 1.06 | 0.25 | |
|  |  |  |  |  |  |  |  |  |  |  |  |  | Eyes closed OSI (20 min) | 2.89 ± 0.98 | 3.91 ± 0.86 | 1.08 | |
| Ely et al. (2012a) [67], U.S. | 32 M; unacclimated | 22±4 | 85.4±10.8 | NS | EX (walking 1.34m/s); 50°C; 3 h work/rest cycle |  | |  |  |  | |  | 20 s dynamic balance test, 10°C |  |  |  | |
|  | Cohort 1, *n* = 8 | | | | | 4.1 | | Concurrent  Rh & Dh | 0.05% NaCl; 100%; 3 h | - | | 90 min  (recovery snack) |  |  |  |  | |
|  |  |  |  |  |  |  |  |  |  |  |  |  | OSIy | 6.6 ± 4.1 | 5.9 ± 4.0 | 0.16 | |
|  |  |  |  |  |  |  |  |  |  |  |  |  | Mean deflection[[14]](#footnote-15) | 5.3 ± 3.6 | 4.6 ± 3.5 | 0.19 | |
|  |  |  |  |  |  |  |  |  |  |  |  |  | Time spent stable | 13.1 ± 6.7 | 15.0 ± 5.6 | 0.29 | |
|  | Cohort 2, *n* = 8 | | | | | 4.2 | | Concurrent  Rh & Dh | 0.05% NaCl; 100%; 3 h | - | | 90 min  (recovery snack) | 20 s dynamic balance test, 20°C |  |  |  | |
|  |  |  |  |  |  |  |  |  |  |  |  |  | OSI | 3.6 ± 0.9 | 2.9 ± 0.8 | 0.78 | |
|  |  |  |  |  |  |  |  |  |  |  |  |  | Mean deflection | 2.6 ± 0.8 | 2.2 ± 0.7 | 0.50 | |
|  |  |  |  |  |  |  |  |  |  |  |  |  | Time spent stable | 17.9 ± 2.1 | 19.2 ± 1.3 | 0.70 | |
|  | Cohort 3, *n* = 8 | | | | | 4.0 | | Concurrent  Rh & Dh | 0.05% NaCl; 100%; 3 h | - | | 90 min  (recovery snack) | 20 s dynamic balance test, 30°C |  |  |  | |
|  |  |  |  |  |  |  |  |  |  |  |  |  | OSI | 3.7 ± 1.6 | 3.0 ± 1.0 | 0.50 | |
|  |  |  |  |  |  |  |  |  |  |  |  |  | Mean deflection | 2.7 ± 1.1 | 2.2 ± 0.9 | 0.47 | |
|  |  |  |  |  |  |  |  |  |  |  |  |  | Time spent stable | 18.0 ± 1.8 | 18.7 ± 1.4 | 0.41 | |
|  | Cohort 4, *n* = 8 | | | | | 4.1 | | Concurrent  Rh & Dh | 0.05% NaCl; 100%; 3 h | - | | 90 min  (recovery snack) | 20 s dynamic balance test, 40°C |  |  |  | |
|  |  |  |  |  |  |  |  |  |  |  |  |  | OSI | 3.6 ± 1.0 | 3.6 ± 1.1 | <0.01 | |
|  |  |  |  |  |  |  |  |  |  |  |  |  | Mean deflection | 2.2 ± 0.9 | 2.6 ± 0.8 | 0.44 | |
|  |  |  |  |  |  |  |  |  |  |  |  |  | Time spent stable | 17.8 ± 1.9 | 17.9 ± 2.5 | 0.04 | |

**Table S7.** Characteristics of research studies evaluating cognitive performance and mood

| **Citation, location** | **Subjects (*n*)** | **Age (y)** | **Pre- Dh BM (kg)** | **VO2 max (mL/kg/min)** | **Dh protocol; temperature; RH; duration** | **BM loss (%)** | **REC 1**  **duration** | | **Rh fluid; % fluid losses replaced; drink time** | **Mean drink rate (mL/h)** | **REC 2 duration** | | **Cognitive domains assessed; temperature; RH; airflow (if applicable)** | **Performance** |
| --- | --- | --- | --- | --- | --- | --- | --- | --- | --- | --- | --- | --- | --- | --- |
| Cian et al. (2001a) [55], France. | 7 M; unacclimated endurance trained | 25 ± 4 | 74.0 ± 4.3 | 58.0 ± 6.7 | EX (~65% VO2 max); 25°C; 35-45%; 120 min | 2.7 | | 60 min | CHO + electrolyte; 100% (1801 ± 267mL); 60 min  (Dh trial: Equal CHO +100mL water) | 1800 | | Testing  20 min & 2h post-Dh | Memory  Image recall  Numerical sequencing  Perceptive discrimination  Judgement of line length  Psychomotor function/processing speed  Two-choice visual reaction task  Mood | The number images of correctly recalled 2 h post-Dh was increased with fluid intake. |
| Cian et al. (2001b) [55], France | 7 M; unacclimated endurance trained | 25 ± 4 | 74.0 ± 4.3 | 58.0 ± 6.7 | HT; 45-50°C; 20-50%; 120 min. | 2.6 | | 60 min | CHO + electrolyte; 100% (1801 ± 267mL); 60 min  (Dh trial: Equal CHO +100mL water) | 1800 | | Testing  20 min & 2h post-Dh h | Memory  Image recall  Numerical recall  Perceptive discrimination  Judgement of line length  Psychomotor function/processing speed  Two-choice reaction task  Mood | The number images of correctly recalled was 2 h post-Dh increased with fluid intake.  Subjective fatigue decreased with fluid intake. |
| Grego et al. (2004) [48], France | 8 M; endurance trained cyclists | 31 ± 7 | 74.0 ± 4.3 | 62.7 ± 6.0 | EX (~65% VO2 max); 20-21°C; 50%; 180 min. | 4.1 | | Concurrent  Rh & Dh | Water (17-20°C); % replaced NS (2200mL); 180 min | 733 | | 5 min | Perceptual discrimination  Critical flicker fusion threshold  Memory/processing speed  Map recognition | No effect |
| Serwah et al. (2006a) [68], Australia | 8 M | 25 ± 3 | 77.6 ± 14.1 | 46.4-60.6 | EX (~70% PPO); 31°C; 63%; max. 90 min. | 1.7 | | Concurrent  Rh & Dh | Water (4.5°C); 100%; max. 90 min | NC | | ~3 min | Psychomotor function/processing speed  One-choice visual reaction task  Two-choice visual reaction task  Four-choice visual reaction task | No effect[[15]](#footnote-16) |
| Serwah et al. (2006b) [68], Australia | 8 M | 25 ± 3 | 77.6 ± 14.1 | 46.4-60.6 | EX (~70% PPO); 31°C; 63%; max. 90 min. | 1.7 | | Concurrent  Rh & Dh | Water (4.5°C); 50%; max. 90 min | NC | | ~3 min | Psychomotor function/processing speed  One-choice visual reaction task  Two-choice visual reaction task  Four-choice visual reaction task | No effect23 |
| Edwards et al. (2007b) [58], N.Z. | 11 M; moderately active soccer  players | 24 ± 3 | 74.0 ± 10.5 | 50.9 ± 4.0 | EX (soccer match + 90% ventilator threshold); 19-25°C; 46-57%; 90 min | 2.4 | | Concurrent  Rh & Dh | Water (20-24°C); 80%; 90min | - | | NS | Visual scanning/processing speed  Sequential number search | No effect |
| Adam et al. (2008a) [56], U.S. | 8 (6M); physically active soldiers | 24 ± 6 | 72.9 ± 11.1 | 48 ± 9 | HT; 45°C; 50%; 3 h | 3.0 | | Concurrent  Rh & Dh | Fluid NS; % replaced NS (BM deficit was 0.3% post-Rh); 3 h  (Dh trial: snack +200mL water) | NC | | 2 h | Psychomotor function/processing speed  Target detection latency  Response latency  Psychomotor function  Shooting accuracy  Perceptive discrimination  Friend-foe discrimination  Visual scanning/vigilance  Target detection  (Thermoneutral environment: 20°C; 50%; 1m/s) | No effect |

| Adam et al. (2008b) [56], U.S. | 8 (6M); physically active soldiers | | 24 ± 6 | | 72.9 ± 11.1 | | 48 ± 9 | | HT; 45°C; 50%; 3 h | | 3.0 | | Concurrent  Rh & Dh | | Fluid NS; % replaced NS (BM deficit was 0.3% post-Rh); 3 h  (Dh trial: snack +200mL water) | | NC | | 2 h | | Psychomotor function/processing speed  Target detection latency  Response latency  Psychomotor function  Shooting accuracy  Perceptive discrimination  Friend-foe discrimination  Visual scanning/vigilance  Target detection  (Cold environment: 2°C; 50%; 2.2m/s) | | No effect | |
| --- | --- | --- | --- | --- | --- | --- | --- | --- | --- | --- | --- | --- | --- | --- | --- | --- | --- | --- | --- | --- | --- | --- | --- | --- |
| D’Anci et al. (2009a) [73], U.S. | | 16 M; university athletes | | 20 ± 1 | | NS | | NS | | EX; 60 min | | 2.0 | | Concurrent  Rh & Dh | | Water; % replaced NS (BM increased 0.1% post-Rh); 60 min | | NC | | NS | | Memory  Digit Span Forward task  Psychomotor function/processing speed  One-choice visual reaction task  Two-choice visual reaction task  Arithmetic/processing speed  Mathematical addition  Visual scanning/vigilance  Number-letter sequence detection  Spatial processing  Mental rotation of 3D objects  Mood | | Two choice reaction time was decreased with fluid intake.  Subjective anger, fatigue, depression, tension and confusion was decreased with fluid intake, whilst vigour increased. |
| D’Anci et al. (2009b) [73], U.S. | | 13 (0 M); university athletes | | 21 ± 1 | | NS | | NS | | EX; 60-75 min | | 1.7 | | Concurrent  Rh & Dh | | Water; % replaced NS (BM deficit 0.2% post-Rh); 60-75 min | | NC | | NS | | Memory  Digit Span Forward task  Psychomotor function/processing speed  One-choice visual reaction task  Two-choice visual reaction task  Response accuracy  Arithmetic/processing speed  Mathematical addition  Visual scanning/vigilance  Number-letter sequence detection  Spatial processing  Mental rotation of 3D objects  Mood | | Two choice reaction time was decreased with fluid intake.  Subjective anger, fatigue, depression, tension and confusion was decreased with fluid intake, whilst vigour increased. |
| Ganio et al. (2011) [69], U.S. | | 24 M; physically fit | | 20 ± 0 | | 79.7 ± 12.1 | | NS | | EX (walking 5.6 km/h); 28°C; 42%; 3x40 min bouts (12:00, 14:00 & 15:00) | | 1.6 | | Concurrent  Rh & Dh | | Water; Est. >100% (BM deficit was 0.10% post-Rh); ~40 min  (Dh trial: 50mL water upon completing each cognitive testing battery) | | NC | | Testing  20 min post each EX bout  (snack at 10:00, 13:50 and 15:50) | | Psychomotor function/processing speed  One-choice visual reaction task  Four-choice visual reaction task  Response latency & accuracy (visual scanning)  Visual scanning/vigilance  Stimuli detection (visual scanning)  Memory/processing speed  Match to sample  Learning/memory  Repeated acquisition  Logical reasoning/processing speed  Grammatical reasoning  Mood  (Thermoneutral environment 23°C) | | Response accuracy (visual scanning) increased and response time (match to sample) was decreased with fluid intake.  Subjective tension/anxiety and fatigue/inertia was decreased with fluid intake. |
| Ely et al. (2012b) [67], U.S. | | 32 M unacclimated | | 22±4 | | 85.4±10.8 | | NS | | EX (walking 1.34m/s); 50°C; 3 h work/rest cycle | | ~4.1 | | Concurrent  Rh & Dh | | 0.05% NaCl; 100%; 3 h | | NC | | 90 min  (recovery snack) | | Psychomotor function/processing speed  One-choice visual reaction task  Four-choice visual reaction task  Memory/processing speed  Match to sample  Logical reasoning/processing speed  Grammatical reasoning  Mood  (Separate cohorts of *n=*8 participants completed cognitive tasks at 10, 20, 30 & 40°C; no effect regardless of environment) | | No effect |
| Wilson et al. (2016) [60], U.K. | | 8 M; licenced jockeys | | 24 ± 3 | | 65.7 ± 7.4 | | NS | | EX (jogging 10km/h), 20°C (wearing sweat suit); 45 min | | 1.8 | | Concurrent  Rh & Dh | | Water; ~100% (700mL); boluses consumed half-way and immediately post-EX | | - | | Est. <15 min | | Response inhibition  Go-No-Go task | | No effect |
| Wittbrodt et al (2015a) [70], U.S. | | 12 M; recreationally active | | 22 ± 2 | | 76.1 ± 1.7 | | 42.8 ± 4.8 | | EX (~60% VO2 max); 32°C; 65%; 50 min | | 1.5 | | Concurrent  Rh & Dh | | Water (9°C); 100% (1180 ± 470mL); 50 min | | 1416 | | NS | | Psychomotor function/processing speed  One-choice visual reaction task  Memory  Match to sample  Perceptive discrimination  Pattern comparison test  Visual scanning/processing speed  Trail making test (parts A and B)  Letter digit substitution  (Hot environment: 32°C; 65%) | | No effect |
| Wittbrodt et al (2015b) [70], U.S. | | 12 M; recreationally active | | 22 ± 2 | | 76.1 ± 1.7 | | 42.8 ± 4.8 | | EX (~60% VO2 max); 32°C; 65%; 50 min | | 1.5 | | Concurrent  Rh & Dh | | Water (9°C); *ad libitum ≈* 80% (940 ± 320mL); 50 min | | 1128 | | NS | | Psychomotor function/processing speed  One-choice visual reaction task  Memory  Match to sample  Perceptive discrimination  Pattern comparison test  Visual scanning/processing speed  Trail making test (parts A and B)  Letter digit substitution  (Hot environment: 32°C; 65%) | | No effect |

**Table S8.** Sensitivity analysis using alternative levels of correlation to complete the meta-analysis, where R = 0.94, 0.84 (actual), 0.74 and 0.50

| ***R*** | **Hedges’ *g* (95% CI)** | ***p* value** | ***I*2 index** |
| --- | --- | --- | --- |
| **Cold + Thermoneutral (≤18°C)** | | | |
| 0.94 | 0.24 (0.13, 0.36) | <0.001 | 68.0 |
| 0.84 | 0.24 (0.12, 0.37) | <0.001 | 62.0 |
| 0.74 | 0.24 (0.12, 0.37) | <0.001 | 59.5 |
| 0.50 | 0.23 (0.11, 0.36) | <0.001 | 56.8 |
| **Warm/Hot (>25**°C) | | | |
| 0.94 | 0.76 (0.64, 0.87) | <0.001 | 14.0 |
| 0.84 | 0.75 (0.61, 0.90) | <0.001 | 7.64 |
| 0.74 | 0.72 (0.57, 0.87) | <0.001 | 0.00 |
| 0.50 | 0.69 (0.53, 0.86) | <0.001 | 0.00 |

**Table S9.** Sensitivity analysis using alternative levels of correlation to complete the meta-regression analysis using the covariates fluid volume, temperature and exercise duration, where R = 0.94, 0.84 (actual), 0.74 and 0.50.

| **Covariate** | **Coefficient (95% CI)** | ***p* value** | **R2** |
| --- | --- | --- | --- |
| **R = 0.94** | | | |
| Fluid volume | 0.003 (-0.003, 0.008) | 0.34 | 0.82 |
| Temperature | 0.025 (0.015, 0.036) | <0.01 |
| Exercise duration | 0.009 (-0.003, 0.021) | 0.11 |
| Exercise protocol | -0.138 (-0.406, 0.130) | 0.28 |
| **R = 0.84 (actual)** | | | |
| Fluid volume | 0.002 (-0.005, 0.009) | 0.56 | 0.95 |
| Temperature | 0.025 (0.015, 0.035) | <0.01 |
| Exercise duration | 0.011 (-0.001, 0.022) | 0.05 |
| Exercise protocol | 0.224 (-0.102, 0.550) | 0.16 |
| **R = 0.74** | | | |
| Fluid volume | 0.001 (-0.006, 0.009) | 0.72 | 0.96 |
| Temperature | 0.025 (0.015, 0.034) | <0.01 |
| Exercise duration | 0.011 (0.001, 0.022) | 0.05 |
| Exercise protocol | -0.241 (-0.618, 0.137) | 0.19 |
| **R = 0.50** | | | |
| Fluid volume | <0.001 (-0.010, 0.010) | 0.930 | 0.95 |
| Temperature | 0.024 (0.015, 0.035) | <0.01 |
| Exercise duration | 0.011 (-0.001, 0.022) | 0.060 |
| Exercise protocol | -0.239 (-0.7331, 0.256) | 0.312 |

1. The maximal multistage running test (MMRT) involved repeated 20 m runs between two points, at increasing intensity. [↑](#footnote-ref-2)
2. The intermittent sprint test (IST) comprised of five 5 s sprints at 3 min intervals [↑](#footnote-ref-3)
3. The maximal anaerobic running test (MART) involved repeated 20 sec runs on a treadmill, at increasing intensities, with 100 sec passive recovery between runs until volitional exhaustion. [↑](#footnote-ref-4)
4. The Yo-Yo Intermittent Recovery Test is a soccer-specific performance test that comprises of 20 m shuttle runs separated by 10 s jog recovery. Running speed during the test is incremental and maximal performance is indicated by total distance covered. [↑](#footnote-ref-5)
5. The intermittent sprint test (IST) comprised of a 36 min of repeated sprint exercise divided into 2 min periods of a 4 sec sprint and 100 s of active recovery (35% VO2 max) and 16 sec passive rest. A repeated sprint bout (RSB) involving 5x2 sec sprints with 18 s active recovery was also completed after the 8th and 16th sprints (RSB 1 and RSB 2). [↑](#footnote-ref-6)
6. The intermittent sprint test (IST) comprised of a 36 min of repeated sprint exercise divided into 2 min periods of a 4 sec sprint and 100 s of active recovery (35% VO2 max) and 16 sec passive rest. A repeated sprint bout (RSB) involving 5x2 sec sprints with 18 s active recovery was also completed after the 8th and 16th sprints (RSB 1 and RSB 2). [↑](#footnote-ref-7)
7. The intermittent sprint test (IST) comprised of a 3 min warm up followed by 6 x 15 s maximal sprints separated by 30 s active recovery [↑](#footnote-ref-8)
8. The Yo-Yo Intermittent Recovery Test is a soccer-specific performance test that comprises of 20 m shuttle runs separated by 10 s jog recovery. Running speed during the test is incremental and maximal performance is indicated by total distance covered. [↑](#footnote-ref-9)
9. Total repetitions in full body resistance exercise protocol: 3 sets of bench press, lat pull down, overhead press, barbell curl, triceps and leg press exercise at 12 RM [↑](#footnote-ref-10)
10. A score of 0 was assigned for a delivery hitting a target. Deliveries that deviated from the target were assigned a score on a scale of 1-6 (line) or 1-10 (length) [↑](#footnote-ref-11)
11. During the Loughborough Passing Test, participants completed a random sequence of eight short and long passes of a soccer ball towards a target, as quickly as possible with the fewest time penalties. In the Loughborough Shooting Test, participants were required to sprint ~12 m, then pass, control and shoot the ball at targets within the goal area. [↑](#footnote-ref-12)
12. The “Ghosting Test” is a squash-specific movement test. Participants were instructed to collect a half-ball that was placed on three racquets positioned around the court, move to the “T”, and then to the next racquet at the opposite corner as quickly as possible. [↑](#footnote-ref-13)
13. The overall stability index (OSI) is an indicator of a subject’s ability to balance on a platform. A higher OSI indicates poorer balance performance. [↑](#footnote-ref-14)
14. Mean deflection was defined as the average position of the subject during the balance test. A higher mean deflection indicates more displacement and poorer balance performance. [↑](#footnote-ref-15)
15. Full results unpublished, findings confirmed with authors [↑](#footnote-ref-16)
